# Prognostic significance of the MELK/TMPO-AS1/hsa-let-7b-5p network in lung adenocarcinoma

**DOI:** 10.3389/fonc.2026.1956981

**Published:** 2026-08-31

**Authors:** Kritika Jangir, Niranjan Kumar, Chainsee Saini, Prerna Vats, Bhavika Baweja, Prachi Patidar, Rashi Soni, Husen Lakdawala, Jaikee Kumar Singh, Rajeev Nema

**Affiliations:** 1 Department of Biosciences, Manipal University Jaipur, Jaipur, Rajasthan, India; 2 Department of Bioinformatics, Marwadi University, Rajkot, Gujarat, India

**Keywords:** ceRNA network, FOXM1, immune modulation, LUAD, MELK

## Abstract

**Introduction:**

Maternal Embryonic Leucine Zipper Kinase (MELK) is a key regulator of the G2/M checkpoint and a recognized pan-cancer oncogene; however, its regulatory mechanisms and clinical significance in lung adenocarcinoma (LUAD) remain incompletely understood. This study aimed to investigate the molecular, prognostic, immune, and therapeutic relevance of MELK in LUAD.

**Methods:**

An integrated multi-omics approach was employed, incorporating gene-expression, survival, transcriptomic, immune-infiltration, regulatory-network, molecular docking, and molecular dynamics analyses. The potential MELK-associated ceRNA regulatory axis was investigated using bioinformatic approaches and subsequently evaluated by qRT-PCR in lung cancer cell lines. The therapeutic potential of candidate MELK-binding compounds was further explored using molecular docking and molecular dynamics simulations.

**Results:**

MELK was markedly overexpressed in LUAD (*log_2_FC = 4.19) and was significantly associated with poor overall survival (HR = 1.63), with stronger prognostic associations in patients with stage I disease (HR = 2.07) and female smokers (HR = 1.50). MELK exhibited a strong positive correlation with FOXM1 (R = 0.834), supporting its coordinated involvement in the G2/M regulatory program. High MELK expression was associated with reduced effector immune-cell infiltration and increased enrichment of exhausted CD8^+^ T cells and regulatory T cells. Integrated regulatory analyses identified a putative **TMPO-AS1/hsa-let-7b-5p/MELK/FOXM1* ceRNA network characterized by increased TMPO-AS1 and MELK expression and reduced hsa-let-7b-5p expression. These expression patterns were further supported by qRT-PCR analysis in lung cancer cell lines. Molecular docking and molecular dynamics simulations identified *hesperidin* as a candidate MELK-binding compound with favorable predicted binding affinity and stable complex behavior.

**Conclusion:**

These findings provide an integrated view of MELK dysregulation in LUAD, linking its G2/M-associated activity with post-transcriptional regulation, immune features, and potential therapeutic targeting. The *TMPO-AS1/hsa-let-7b-5p/MELK/FOXM1* axis may represent a promising molecular framework for understanding MELK-mediated LUAD progression and identifying prognostic biomarkers and therapeutic opportunities.

## 1 Introduction

Lung cancer accounts for a large share of cancer deaths worldwide (1). Non- small cell lung cancer (NSCLC) is the most common type, including lung adenocarcinoma (LUAD) and lung squamous cell carcinoma (LUSC). LUAD arises from mucus- secreting cells in the distal airways, whereas LUSC arises from the bronchial epithelium (2). LUAD histological subtypes include lepidic- predominant (40–60%), acinar (40–50%), and papillary (5–10%), which are generally less aggressive and associated with a favorable prognosis. The more invasive and metastatic subtypes include solid- predominant (20–30%), mucinous (2–10%), micropapillary (2–5%), clear cell (1–2%) and signet ring cell (1–4%) (3). The high incidence, exceeding two million new cases annually, is driven largely by tobacco smoke (about 85%) and environmental exposures such as air pollution (4). Diagnosis typically integrates imaging (chest X-ray, CT, PET- CT) with histopathology, biopsy, immunohistochemistry, and molecular profiling (5). Oncogenic drivers such as EGFR, ALK, KRAS, and ROS1 have enabled targeted therapies and personalized treatment (6). Despite advances, many cancers are detected at advanced stages, limiting treatment efficacy (7). Treatments include surgery, chemotherapy, targeted therapies, and immune checkpoint inhibitors, which have extended survival but remain limited by toxicity, resistance, and tumor heterogeneity. These limitations highlight the need for a deeper understanding of the molecular mechanisms underlying lung cancer progression. Dysregulation of the cell cycle is a hallmark of lung carcinogenesis, with MELK playing a role in cell- cycle regulation (8). Physiologically, MELK regulates mitotic progression and G2/M checkpoint control in embryonic and progenitor cells (9). MELK contains an N-terminal kinase domain that mediates its phosphorylation-dependent regulatory functions and interacts with key mitotic regulators to coordinate cell-cycle progression (10). In tumors, especially LUAD, MELK is overexpressed, promoting proliferation via the PLK1-CDC25C-CDK1 axis, disrupting checkpoints, and contributing to genomic instability and resistance to apoptosis (11). Mechanistically, the MELK–PLK1–CDC25C–CDK1 cascade facilitates mitotic entry and progression, thereby supporting continuous cell division and allowing tumor cells to overcome normal G2/M checkpoint control (11). Dysregulation of this pathway may consequently contribute to chromosomal instability and persistent tumor-cell proliferation. MELK is also associated with EMT and immune evasion, modulating immune signaling to suppress antitumor immunity (11, 12). A functional MELK–FOXM1 axis may drive sustained proliferative signaling and tumor progression (13). FOXM1 is a central transcriptional regulator of the G2/M program and controls the expression of multiple genes required for mitotic progression (14). The functional association between MELK and FOXM1 therefore provides a mechanistic link between MELK activity and sustained activation of the mitotic transcriptional program, potentially reinforcing uncontrolled proliferation and tumor progression (13). Alongside its role in cell-cycle regulation, MELK has also been implicated in cellular responses to DNA damage and therapeutic stress (15). Increased MELK activity may enhance tumor-cell survival under genotoxic conditions, whereas MELK inhibition has been associated with increased DNA damage and reduced cellular viability (16). These observations suggest that MELK may contribute to resistance to DNA-damaging therapies and radiotherapy, supporting its potential as a therapeutic sensitization target (17). At the post-transcriptional level, ceRNA networks regulate cancer progression by allowing lncRNAs and mRNAs to compete for microRNAs, influencing proliferation, metastasis, and immune modulation (18). Targeting ceRNA regulation of MELK could suppress oncogenic signaling and restore cell- cycle control. Integrating ceRNA modulation with small-molecule inhibition may selectively target MELK-driven oncogenesis, reduce toxicity and improve efficacy in lung cancer. In this article, we discuss an integrated approach to validate MELK-associated ceRNA in lung cancer.

## 2 Methodology

### 2.1 MELK chromosomal mapping, functional annotation, and pan-cancer expression analysis

The chromosomal localization of the MELK gene was identified using the GeneLoc (19). Next, functional annotation was conducted through retrieval of GO terms from the public database, GeneCards (20), to determine MELK’s roles in various cellular pathways. To assess MELK expression across cancer types, differential gene expression data were obtained from the OncoMX (21), and log_2_ fold change (log_2_FC) and statistical significance values were extracted. Simultaneously, TIMER 2.0 (22), GSCA (23), and UALCAN platforms (24) were employed to validate the MELK expression levels across TCGA cancers.

### 2.2 Prognostic analysis and expression profiling of MELK in LUAD

The Kaplan-Meier plotter (KMP) database (25) was used to associate MELK gene expression levels with survival outcomes in lung cancer patients. Patients were divided into low- and high-MELK expression cohorts based on the median expression value. Comprehensive survival analyses were conducted using multiple univariate and multivariate parameters to strengthen the findings. MELK (Affy ID: 204825_at) expression was evaluated across different survival metrics, including Overall Survival (OS), validated by using the miRtarget (26) database, followed by evaluation of, First Progression Survival (FP), Post-Progression Survival (PPS), and OS stratified by histological subtypes, LUAD and LUSC. Further stratification included analysis of various clinical variables such as cancer stage, gender, and smoking history in LUAD patients to explore potential associations between MELK expression and patient prognosis in specific subgroups. Only results meeting the inclusion criteria (p-value < 0.05 and HR > 1) were considered statistically significant and indicative of poor prognosis, whereas those not meeting these thresholds (p-value> 0.05 or HR < 1) were excluded based on the exclusion criteria.

Further, the survival analysis of MELK protein expression was also evaluated using the Human Protein Atlas (HPA) (27). Additionally, to comprehensively analyze the differential gene expression (DGE) pattern of MELK in normal vs. LUAD samples, a multi database bioinformatics approach was employed, such as UALCAN, ENCORI (28), OncoDB (29), GEPIA2 (30), and Lung Cancer Explorer (LCE) (31). Using R-based programs, MELK expression data for LUAD were obtained from the TCGA-LUAD dataset with sample normalization, maintaining an equivalent number of tumor (n = 59) and normal (n = 59) patients. Furthermore, MELK expression at the single-cell transcriptomic level was analyzed using TCGAnalyzeR (32). A high-throughput single-cell transcriptomic database was used to construct a volcano plot to identify significantly dysregulated transcripts, with MELK emerging among the top upregulated genes (log2FC > 1). Genes with a fold change >1 were classified as upregulated, and those <1 were downregulated. Further stratified analyses were conducted using the UALCAN database to assess MELK expression across tumor stages, smoking status, and clinicopathological features in LUAD patients. The GSCA database was also used to validate the stage-wise expression trend of MELK relative to MKI67. The LCE database was used to determine the expression pattern of MELK across female smoker and non-smoker datasets.

### 2.3 Analysis of MELK-associated biological processes, heterogeneous model and transcription factor

The CancerSEA database (33) was used to explore MELK’s involvement in cellular processes, including cell cycle, proliferation, DNA repair, invasion, and EMT. For all analysis, results were considered significant (included) when p < 0.05, whereas non-significant findings (p > 0.05) were excluded from further interpretation. To study the correlation between MELK expression and TP53 mutation, UALCAN data were analyzed and further validated using TIMER 2.0. We examined the correlation of MELK with cell cycle regulators and EMT markers using the ENCORI database, while TNMplot (34) was used to compare MELK expression in normal, tumor, and metastatic samples. Further, to uncover potential transcriptional regulators of MELK in LUAD, we employed an integrative, stepwise bioinformatics approach. Using the UALCAN database, we identified genes whose expression levels were positively and negatively correlated with MELK in LUAD samples. To ensure these associations were statistically reliable, we validated the correlations using independent datasets accessed through the ENCORI platform. Next, to investigate how these MELK-associated genes might interact functionally, we constructed a protein-protein interaction (PPI) network using the STRING database (35), thereby identifying groups of genes involved in shared biological processes. To explore regulatory relationships and directionality within this network, the SIGNOR signaling database (36) was used to identify candidate transcription factors upstream of MELK. We then evaluated correlations between MELK and these candidates across ENCORI, OncoDB, and GEPIA2 to confirm consistency prior to downstream analyses. We then assessed the co-expression relationships between these transcription factors and MELK through detailed correlation analyses in ENCORI. To evaluate the potential clinical impact of these regulators, we conducted survival analyses using the KMP tool across both overall lung cancer and LUAD-specific cohorts. To confirm whether the expression of these candidate transcription factors was altered in tumors, we performed differential expression analyses using ENCORI, UALCAN, and custom R-based pipelines, and validated our findings at the transcriptomic level using TCGAnalyzeR. We also explored expression patterns across tumor stages and smoking status groups using UALCAN, GSCA, and the LCE database. Furthermore, we examined the correlation between FOXM1 and key cell-cycle regulators, as well as EMT-associated markers, using ENCORI, given its potential role as an upstream regulator within the MELK-associated network.

### 2.4 Tumor immune microenvironment associated with MELK

To investigate the role of MELK and its transcriptional regulator FOXM1 in modulating the tumor immune microenvironment (TIME) of NSCLC, a comprehensive integrative analysis was performed using both bulk and single-cell transcriptomic datasets. Initially, immune expression profiling was conducted using the Spring viewer platform (37) to visualize the distribution of MELK and FOXM1 across major immune cell populations. To quantitatively assess the predictive capacity of these genes for immune responsiveness, receiver operating characteristic (ROC) curve analyses were performed using TIMER 3.0 (38), and area under the curve (AUC) values were computed for both MELK and FOXM1. Subsequently, TIMER 2.0 was employed to evaluate the correlation between MELK expression and immune infiltration levels across LUAD samples. To further enhance these findings, MELK-associated positively and negatively correlated cell cycle regulators were examined for their association with immune cell populations. This comparison identified key immune subsets that consistently aligned with the MELK regulatory axis, highlighting its putative role in modulating tumor-associated immunity. Immune cell infiltration analysis for TCGA-LUAD samples was obtained directly from the GSCA (Gene Set Cancer Analysis) database. The correlations were visualized as a heatmap, with colors indicating positive or negative associations. Statistical significance was evaluated using Spearman’s rank correlation test, with symbols denoting significant correlations. Further, to achieve single-cell resolution, CellTracer (Q. Guo et al., 2023) was used with the NSCLC_EMTAB6149 dataset, which encompasses both tumor and immune compartments. The analysis involved class-based and immune-specific UMAP clustering, followed by mapping of MELK expression across clusters. Mean expression per cell type was quantified, and a cut-off threshold of 0.02 was applied to select the most enriched populations. Finally, to understand developmental dynamics, pseudotime trajectory analysis was performed using Monocle 2. Cluster and cell type trajectories were reconstructed, and MELK expression was visualized along the pseudotime path.

### 2.5 Molecular docking and interaction profiling of MELK

We selected the MELK direct inhibitor OTSSP167 as a reference compound because of its ability to directly target the ATP-binding pocket of MELK (11). Then, we extended the analysis to a focused panel of G2/M checkpoint-targeting drugs, as they inhibit WEE1, CHK1/CHK2, PLK1, Aurora kinases, MYT1, and microtubule dynamics regulators, which govern mitotic entry, spindle formation, and progression through checkpoint control cellular contexts in which MELK activity becomes critically essential. Importantly, the selected G2/M inhibitors have undergone preclinical evaluation and have progressed through different phases of clinical trials, either as monotherapies or in combination. However, their clinical use is often associated with side effects like high toxicity to healthy cells, poor selectivity, and the potential to induce adverse events in patients, underscoring the need for safer yet therapeutically effective agents (39). This key limitation motivated us to investigate flavonoids due to their ability to modulate kinase-associated signaling pathways, cell cycle regulation, and mitotic stress responses, together with their natural origin, lower toxicity profiles, well-documented anticancer and antioxidant properties, and structural compatibility with ATP binding sites (40, 41). Specifically, flavanone glycosides (naringin, neohesperidin, hesperetin, narirutin, eriocitrin, and hesperidin) and flavonols (kaempferol, myricetin, rutin, isorhamnetin, fisetin, and quercetin) were selected to represent closely related but chemically distinct polyphenolic scaffolds, enabling assessment of how differences in ring saturation, glycosylation, and hydroxylation patterns, key determinants of kinase ligand interactions, affect accommodation within the MELK’s ATP binding pocket.

Further, the 3D crystal structure of human MELK (PDB ID: 5TWL) was retrieved from the Protein Data Bank (PDB) and visualized using Discovery Studio Visualizer (DSV). 2D interaction diagrams were generated to identify residues involved in hydrogen bond formation within the kinase domain. To further characterize the interaction landscape, protein-ligand interaction profiling was performed using the protein-ligand interaction profiler (PLIP) server, enabling systematic identification of hydrogen bonding and other noncovalent interactions. In parallel, the full-length MELK protein sequence was examined using NCBI resources to locate the conserved ATP binding pocket, which is critical for kinase catalytic activity. Residues consistently identified by structural visualization, interaction profiling, and ATP-binding pocket mapping were used to define the active site region. Based on this integrated residue selection approach, grid box coordinates were determined (X = 4.626, Y = 5.696, Z = -6.091). Ligand structures were retrieved from PubChem, energy-minimized, and prepared using AutoDock Tools. Molecular docking was performed using AutoDock Vina, with the grid box centered on the defined ATP-binding site of MELK. Multiple docking conformations were generated for each ligand, and the top-ranked pose based on binding affinity was selected for interaction analysis. Protein ligand interactions, including hydrogen bonds and other noncovalent contacts, were examined using DSV, and both 2D and 3D interaction diagrams were generated to visualize ligand engagement with key active site residues.

### 2.6 Molecular dynamics simulation

To further validate the stability of the docked MELK–OTSSP167 and MELK–Hesperidin complexes under dynamic physiological conditions, molecular dynamics (MD) simulations were performed using GROMACS (version 26.2) with the CHARMM36 force field. The top-ranked docking poses were used as the initial structures for simulation. Each complex was solvated in a TIP3P water box, neutralized with appropriate counter ions, and subjected to energy minimization followed by NVT and NPT equilibration. Subsequently, a 100 ns production simulation was carried out under periodic boundary conditions. Structural stability and conformational behavior of both complexes were assessed by analyzing root mean square deviation (RMSD), root mean square fluctuation (RMSF), radius of gyration (Rg), solvent-accessible surface area (SASA), and hydrogen bonding using the built-in GROMACS analysis tools.

### 2.7 Identification of MELK–FOXM1-associated ceRNA network

miRNAs targeting MELK and FOXM1 were identified using the miRNet database (42), which predicted a shared regulatory network between both genes. The expression profiles of these miRNAs were analyzed using the UALCAN database to determine differential regulation in LUAD versus normal tissues. Downregulated miRNAs were first screened using UALCAN, and their correlations with MELK and FOXM1 were subsequently analyzed using ENCORI. miRNAs showing negative correlation coefficients and downregulated expression were shortlisted for further evaluation. Survival analysis of selected candidates was performed using the KM Plotter database, and diagnostic accuracy was assessed using ROC analysis to determine AUC values for each miRNA. The binding affinity between shortlisted miRNAs and MELK mRNA was validated using miRwalk (43) and RNA22v2 databases (44), where minimum free energy (MFE) values were noted to confirm stable miRNA-mRNA duplex formation. Selected miRNAs Kaplan–Meier survival analysis using UALCAN (miRtarget) has been done. The final expression validation of miRNAs was conducted using UALCAN, CancerMIRNome (45), and R-based normalization plots. Using TCGA LUAD miRNA expression data, the stagewise expression trend of hsa-let-7b-5p was analyzed across pathological stages using R-based statistical and visualization pipelines.

Additionally, to elucidate the upstream regulatory network of the selected miRNA in LUAD, potential interacting long non-coding RNAs (lncRNAs) were identified utilizing the Enrichr database (46), which integrates experimentally validated and predicted RNA interactions. Among the 97 candidate lncRNAs retrieved, those exhibiting significant differences in LUAD tumor tissues compared to normal lung samples were selected based on analyses conducted via the UALCAN platform. Subsequent correlation analyses were performed using the ENCORI database to assess the relationship between lncRNAs, hsa-let-7b-5p, and the oncogenes MELK and FOXM1. The prognostic significance of these lncRNAs was further evaluated using survival analyses in the KMP database, which integrates clinical and gene expression data from lung cancer cohorts. Additionally, the binding affinity of the selected lncRNA and miRNA was analyzed using the RNA22v2 database.

Further, to assess TMPO-AS1 expression across cancer types, differential gene expression data were obtained from the lncRNAfunc 2.0 database (47). TMPO-AS1 was validated using ENCORI, OncoDB, UALCAN, and LCE were employed to compare TMPO-AS1 expression between LUAD and normal tissues, and the results were validated using R-based normalization plots. The TCGAnalyzeR database was used to validate MELK expression at the transcript level. Stage-wise expression patterns and smoking-based comparisons were evaluated using UALCAN and validated using GSCA and LCE. Correlation analyses were performed to explore the relationships between hsa-let-7b-5p and TMPO-AS1 expression, and between genes involved in cell-cycle regulation and EMT within the MELK-associated network, using the ENCORI database. Lastly, a ceRNA network comprising MELK, FOXM1, hsa-let-7b-5p, and TMPO-AS1 was constructed using the miRNet and Cytoscape (48) databases.

### 2.8 Cell culture

The human lung cancer cell lines A549 and H460 were obtained from AIIMS Bhopal, and H23 was obtained from the National Center for Cell Science (NCCS), Pune, India. The normal human lung fibroblast cell line MRC-5 was obtained from AIIMS, New Delhi, India. A549, H460, and H23 were cultured in Dulbecco’s Modified Eagle Medium (DMEM; Thermo Fisher Scientific, #3256037), whereas MRC-5 was cultured in Roswell Park Memorial Institute (RPMI) medium (Gibco, #3327858) supplemented with 10% fetal bovine serum (FBS; Thermo Fisher Scientific, #2724136) and 1% penicillin-streptomycin (10,000 units/ml penicillin and 10,000 µg/ml streptomycin; Gibco, #2706608). Cells were maintained in a humidified incubator at 37 °C with 5% CO_2_. Subculturing was performed at ~90% confluence using 0.25% trypsin-EDTA (Thermo Fisher Scientific, #25200072).

### 2.9 qRT-PCR

Total RNA was extracted from cells using the PureLink™ RNA Mini Kit (Thermo Fisher Scientific#2896318), while miRNA was isolated using the miRNA Isolation Kit (GeNei, #KT2451053) according to the manufacturer’s instructions. Subsequently, RNA was reverse transcribed into cDNA using the iScript™ cDNA Synthesis Kit (Bio-Rad, #1708891) on a Nexus Gradient Thermal Cycler (Eppendorf). The obtained cDNA was then subjected to quantitative real-time PCR (qPCR) using iTaq™ Universal SYBR^®^ Green Supermix (Bio-Rad, #1725121) on a CFX96 Real-Time PCR Detection System (Bio-Rad). The specific primer sequences used in this study are mentioned in **Supplementary Table 21**. U6 was used as the internal control for normalization of hsa-let-7b-5p, while glyceraldehyde-3-phosphate dehydrogenase (GAPDH) was used as the internal control for MELK and TMPO-AS1. Relative expression levels were analyzed using the 2^-ΔΔCt method.

### 2.10 Statistical analysis

To investigate differential expression of MELK, lncRNA TMPO-AS1, and miRNA hsa-let-7b-5p in LUAD, transcriptomic data were retrieved from publicly available in silico gene expression repositories. RNA-seq data for mRNAs and lncRNAs, as well as miRNA expression profiles, were log2-transformed and normalized before statistical evaluation. Expression differences between tumor tissues and adjacent normal samples were assessed using the Wilcoxon rank-sum test, with p-values < 0.05 considered statistically significant. Boxplots illustrating expression levels were generated using the ggplot2 package in R. For bulk RNA-seq data, upregulated regions were included if the log2 fold change (FC) > 1, while downregulated regions with FC < 1 were excluded. All statistical comparisons were performed using the default parameters of each platform, and a p-value < 0.05 was considered statistically significant. qRT-PCR data are presented as mean ± SD. Statistical significance was determined by comparison with the control (MRC-5) group, where *p < 0.05, **p < 0.01, and ***p < 0.001.

## 3 Results

### 3.1 MELK functional annotation and pan-cancer analysis

Given the emerging evidence linking MELK to oncogenic proliferation, therapy resistance, and cancer stemness, we first explored its genomic features and distribution across the human genome to establish a baseline for downstream analyses. The MELK gene was located on chromosome 9 at 9p13.2 (**Supplementary Figure 1A**). MELK encodes a serine/threonine kinase that plays a central role in regulating essential cellular processes. Functional enrichment analysis based on GO (**Supplementary Table 1**) revealed that MELK is involved in multiple critical biological activities, including the G2/M transition of the mitotic cell cycle, protein phosphorylation, chromatin remodeling, and several others. These findings suggest that MELK is a multifunctional protein closely linked to cell division, survival, and oncogenic transformation, making it biologically relevant for further analysis.

Moreover, several previous studies have associated MELK overexpression with poor prognosis across various cancers (49–51); therefore, to assess MELK expression patterns across pan-cancer, we utilized the OncoMX database, which revealed that MELK is significantly upregulated in multiple cancers, as shown in **Table 1**. Notably, the highest expression was observed in lung cancer, with a log2FC of 4.19 and a significant p-value of 2.25e-267. To validate these observations, MELK expression was further examined using multiple databases, including TIMER, GSCA, and UALCAN (**Supplementary Figures 1B–D**). Across all three platforms, MELK expression was consistently and significantly upregulated in lung tumor tissues compared to normal lung tissues. Collectively, these results establish MELK as a recurrently dysregulated gene across diverse malignancies, with lung cancer showing noticeable elevation.

**Table 1:** MELK’s pan cancer analysis.

UniProtKB/SwissProt AC	Gene symbol	Log2 F.C.	P-value	Significant	Expression trend	TCGA study	Source
Q14680	MELK	4.19	2.25e-267	Yes	up	lung cancer	TCGA
		3.89	2.37e-109	Yes	up	uterine cancer	
		3.82	3.7e-78	Yes	up	liver cancer	
		3.63	8.62e-184	Yes	up	breast cancer	
		2.67	4.91e-107	Yes	up	kidney cancer	
		2.38	2.49e-18	Yes	up	bladder cancer	
		2.05	3.49e-14	Yes	up	esophageal cancer	
		1.86	1.05e-25	Yes	up	stomach cancer	
		1.56	4.63e-31	Yes	up	head_and_neck cancer	
		1.54	9.88e-17	Yes	up	prostate cancer	
		1.42	3.13e-48	Yes	up	colorectal cancer	
		0.68	0.0000449	Yes	up	thyroid cancer	

### 3.2 Prognostic significance of MELK expression in lung cancer patients

**Figure 1:**
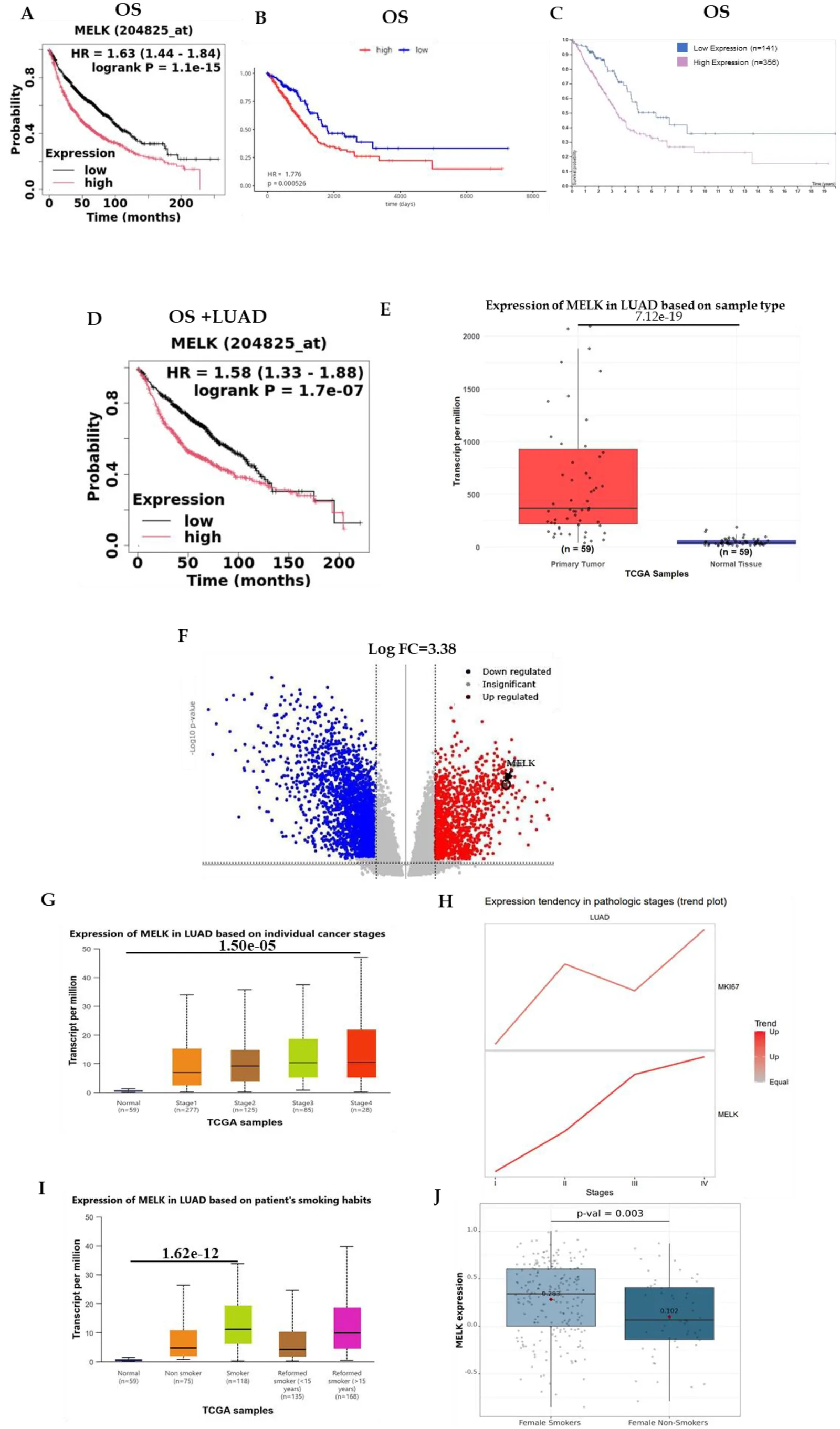
Prognostic and expression profiling of MELK in LUAD. Survival plots showing overall survival (OS) in lung cancer patients using **(A)** KM Plotter, **(B)** UALCAN, **(C)** Human Protein Atlas, and **(D)** KM plotter curve showing OS + LUAD (n = 1161). MELK expression in normal vs. tumor tissue: **(E)** R-based package (normal n = 59, tumor n = 59). **(F)** Transcriptome analysis in LUAD using TCGAnalyzeR. MELK expression based on cancer stages by using **(G)** UALCAN and **(H)** GSCA. **(I)** UALCAN analysis showing MELK expression by smoking status. **(J)** LCE comparative analysis of MELK expression in female smokers vs. non-smoker females.

To evaluate the prognostic relevance of MELK in lung cancer, survival analyses were conducted using the KMP database, integrating microarray data from TCGA and GEO datasets. Patients with high MELK expression exhibited significantly reduced OS compared to those with low expression levels (Hazard Ratio (HR) = 1.63, 95% CI = 1.44-1.84, P = 1.1e-15), as shown in **Figure 1A**, with median survival declining from 95 months in the low-expression cohort to 47.6 months in the high-expression cohort. Further OS analysis, as shown in **Figure 1B**, by UALCAN (miRtarget) confirmed this trend (HR = 1.776, P = 0.000526). Similarly, FP survival using KMP was markedly lower in the high MELK expression cohort (11 months) than in the low expression cohort (31.41 months), with an HR of 1.82 (P = 3.9e-12), highlighting its strong association with aggressive disease progression. Although a similar trend was also observed in PPS for the high-expression group (10.78 vs. 17.67 months), no significant (P = 0.085) result was observed (**Supplementary Table 2**). Interestingly, MELK overexpression was consistently associated with poor OS at the protein level, as visualized in the HPA (**Figure 1C**). Further, when stratified by histological subtype, high MELK expression remained significantly associated with poorer OS in LUAD patients (HR = 1.58, P = 1.7e-07; **Figure 1D**), whereas no significant association was observed in LUSC (HR = 1.03, P = 0.74; **Supplementary Table 2**). This suggests that the prognostic significance of MELK is specific and can be considered as a molecular classifier for the adenocarcinoma subtype.

Stage-wise analysis further indicated that MELK’s adverse prognostic impact is most pronounced in early-stage disease. In stage I LUAD patients, high MELK expression was associated with nearly half the median survival time (40.3 months) compared with low expression (76 months), with an HR of 2.07 (P = 0.00035), suggesting its potential as an early-stage prognostic indicator. However, no significant association was observed in stage II and stage III cohorts (**Supplementary Table 2**). Further gender-based survival trends revealed that both male and female LUAD patients with elevated MELK levels had worse outcomes, with HR of 1.47 (P = 0.0012) and 1.73 (P = 8.1e-05), respectively. Furthermore, MELK expression was significantly associated with poor survival among smokers (HR = 1.54, P = 0.001), whereas no statistically significant value was observed in non-smokers (P = 0.13). Notably, within the smoking cohort, female smokers showed a stronger negative association between MELK expression and survival (HR = 1.50, P = 0.042) compared to male smokers (HR = 1.29, P = 0.15), suggesting a possible relationship between gender, smoking behavior, and MELK-driven oncogenic signaling (**Supplementary Table 2**). Female smokers with MELK overexpression accounted for 19.45% of the total LUAD patients with MELK overexpression. These findings demonstrate that MELK overexpression serves as a powerful predictor of poor prognosis in LUAD, particularly in early-stage tumors and among female smokers. The consistent trends across multiple clinical subgroups highlight MELK as a clinically relevant biomarker and potential therapeutic target in LUAD.

### 3.3 Expression profiling of MELK in lung adenocarcinoma and its clinicopathological status

To understand MELK expression levels in LUAD, several reliable TCGA-based databases were used. As shown in **Supplementary Figures 2A–E**, the UALCAN, ENCORI, OncoDB, GEPIA2, and LCE data showed a significant rise in MELK levels in tumor samples (p < 0.05), confirming its close link with malignancy. Further, validation using R-based packages (TCGA) with normalized sample numbers between normal vs. tumor (N = 59 each), as shown in **Figure 1E**, also showed an increase in MELK expression in LUAD tumor samples compared to normal ones (P = 7.12e-19). Additionally, a volcano plot from TCGAnalyzeR, as shown in **Figure 1F**, showed that MELK transcripts were among the upregulated mRNAs (logFC = 3.38), confirming its dominant expression at the transcriptomic level. These consistent findings across multiple datasets suggest that MELK may act as a key oncogenic driver in LUAD.

Furthermore, when MELK expression was analyzed across different clinical subgroups using UALCAN, MELK levels were high in advancing tumor stages, as shown in **Figure 1G**. Next, when MELK expression was compared to MKI67, a well-known cell proliferation marker, across tumor stages by using GSCA, the trend plot showed a more reliable pattern for MELK than MKI67, as shown in **Figure 1H**, indicating that MELK could serve as an additional, supportive biomarker when used in combination with MKI67, offering improved insight into tumor growth dynamics. Additionally, when patients were grouped by smoking habits, as shown in **Figure 1I**, MELK expression was significantly upregulated in LUAD tissues compared to normal samples and showed a progressive increase with higher nodal metastasis status, as analyzed using UALCAN (**Supplementary Figure 2F****; p-value < 0.001)**. These results suggest that MELK overexpression is associated with metastatic progression in LUAD. The LCE database, as illustrated in **Figure 1J**, also showed that female smokers had higher MELK levels compared to non-smoker females (P = 0.003), indicating that MELK might be influenced by both smoking and gender factors.

### 3.4 MELK as a regulator of tumor progression, EMT, and TP53-associated proliferation in lung adenocarcinoma

**Figure 2:**
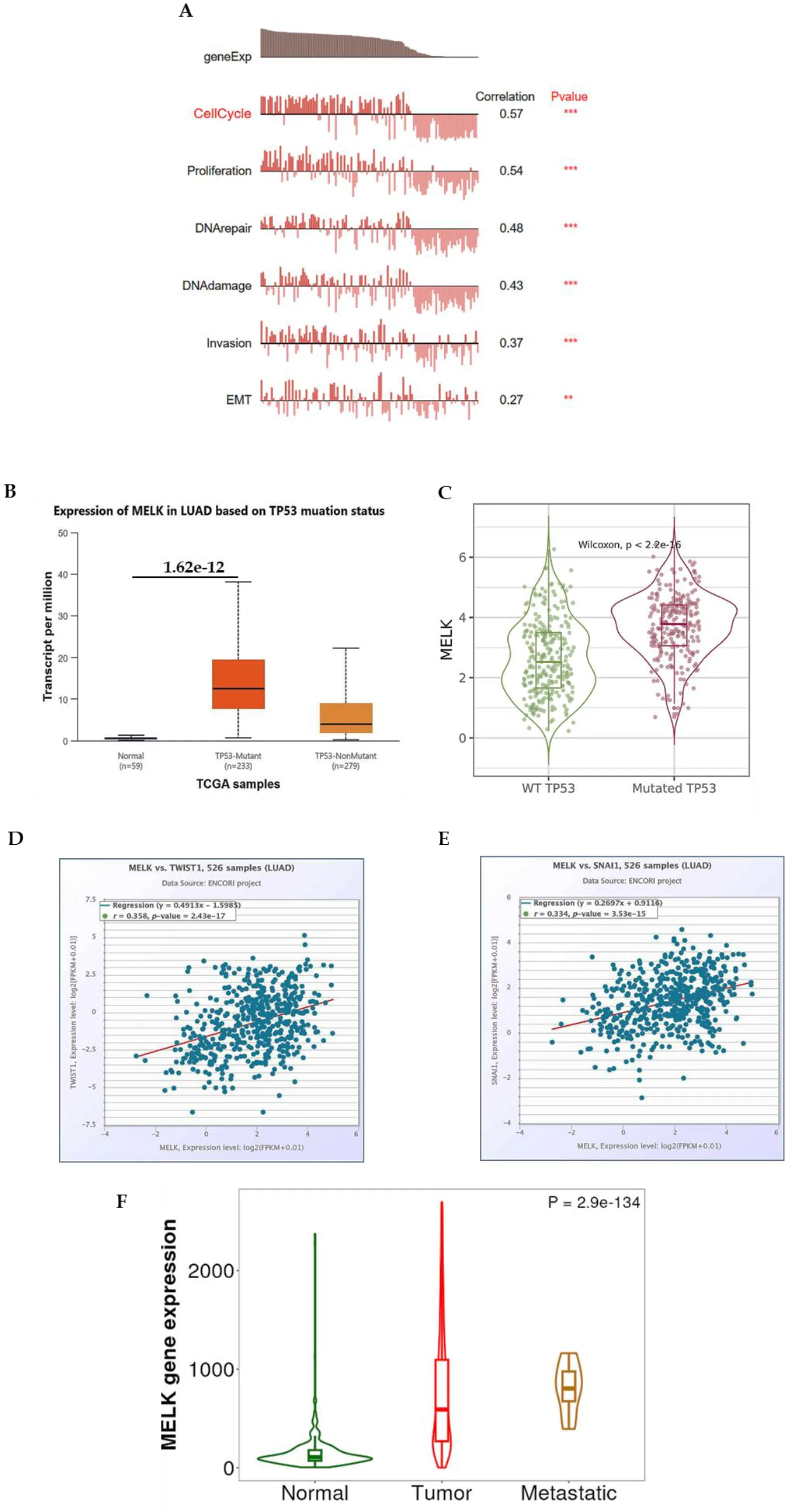
Functional association of MELK with tumor progression and EMT in LUAD. **(A)** CancerSEA showing functional enrichment of MELK in oncogenic processes. Box plot and violin plot showing MELK expression in TP53-mutant vs non-mutant LUAD by using **(B)** UALCAN and **(C)** TIMER 2.0, respectively. ENCORI shows the correlation of MELK with EMT markers **(D)** TWIST1 and **(E)** SNAI1. **(F)** TNM boxplot showing MELK expression from normal and tumor to metastatic LUAD samples.

To further elucidate the oncogenic role of MELK and its regulatory involvement in tumor progression, multiple in silico datasets were analyzed. The CancerSEA database, as shown in **Figure 2A**, revealed that MELK is functionally enriched in critical cellular processes, including cell cycle progression (R = 0.57), proliferation (R = 0.54), DNA repair (R = 0.48), DNA damage response (R = 0.43), invasion (R = 0.37), and epithelial-mesenchymal transition (EMT; R = 0.27), all showing statistically significant correlations. Supporting graphical representations of MELK’s correlation with these cellular processes are provided in **Supplementary Figures 3A–F**. The positive association of MELK with proliferation indicates that MELK plays a vital role in sustaining tumor progression. As already known, the role of TP53 in suppressing tumor proliferation and regulating cell cycle arrest and apoptosis (52), led us to explore whether or not the MELK-driven proliferation is influenced by TP53 mutation status. Analysis using the UALCAN database, as provided in **Figure 2B**, demonstrated significantly higher MELK expression in TP53-mutant LUAD samples compared to non-mutant and normal tissues, suggesting that loss of TP53 function enhances MELK-mediated proliferative signaling. This finding was further validated using TIMER 2.0 (**Figure 2C****),** where MELK expression was markedly elevated in TP53-mutated tumors (P = 2.2e-16), confirming that TP53 mutations may facilitate uncontrolled MELK-associated proliferation in LUAD cells. To determine whether this association extends to TP53 wild-type cases, we examined the correlation between TP53 and MELK expression. Although the correlation was weak (R = -0.0885), the observed inverse relationship suggests that TP53 expression tends to decrease with increasing MELK expression in LUAD.

Furthermore, we evaluated the correlation between MELK expression and key G2/M cell-cycle regulators, including CDK1 (R = 0.865), CCNB1 (R = 0.840), PLK1 (R = 0.836), and CDC25C (R = 0.843). These strong positive correlations indicate a close association between MELK and core G2/M cell-cycle regulators, supporting its involvement in G2/M phase progression (**Supplementary Table 3**). Additionally, we investigated MELK’s contribution to EMT; we performed correlation analyses using the ENCORI platform, focusing on established EMT markers identified in prior studies (53) (**Supplementary Table 4**). As shown in **Figures 2D, E**, MELK expression was positively correlated with both TWIST1 (R = 0.53, P = 2.4e-17) and SNAI1 (R = 0.33, P = 3.3e-15), supporting its role in promoting EMT-related transcriptional reprogramming and enhancing tumor invasiveness. Importantly, both TWIST1 and SNAI1 also demonstrated significant prognostic value in LUAD. In the lung cancer cohort (n = 2166), high expression of TWIST1 and SNAI1 was associated with reduced OS (TWIST1: HR = 1.24, 95% CI: 1.1-1.39, P = 0.00045; SNAI1: HR = 1.27, 95% CI: 1.13-1.43, P = 6.8e-05), with median survival decreasing from 80 to 59.5 months and 78.9 to 57 months, respectively. In LUAD-specific analysis (n = 1161), the prognostic impact was even more pronounced for SNAI1 (HR = 1.37, P = 0.00027), with median survival dropping from 99 to 69.9 months between low- and high-expression groups. TWIST1 also remained statistically significant (HR = 1.21, P = 0.029), indicating that elevated expression of these EMT drivers is associated with worse clinical outcomes in LUAD (**Supplementary Table 5**). Finally, to assess the metastatic potential of MELK, TNMplot analysis (**Figure 2F**) revealed a significant increase in MELK expression from normal to tumor to metastatic LUAD samples (P = 2.9e-134). Collectively, these findings suggest that MELK may act as a key regulator of proliferation, EMT, and metastasis in LUAD.

### 3.5 Identification of a heterogeneous model and the key transcription factor regulating MELK in LUAD

**Figure 3:**
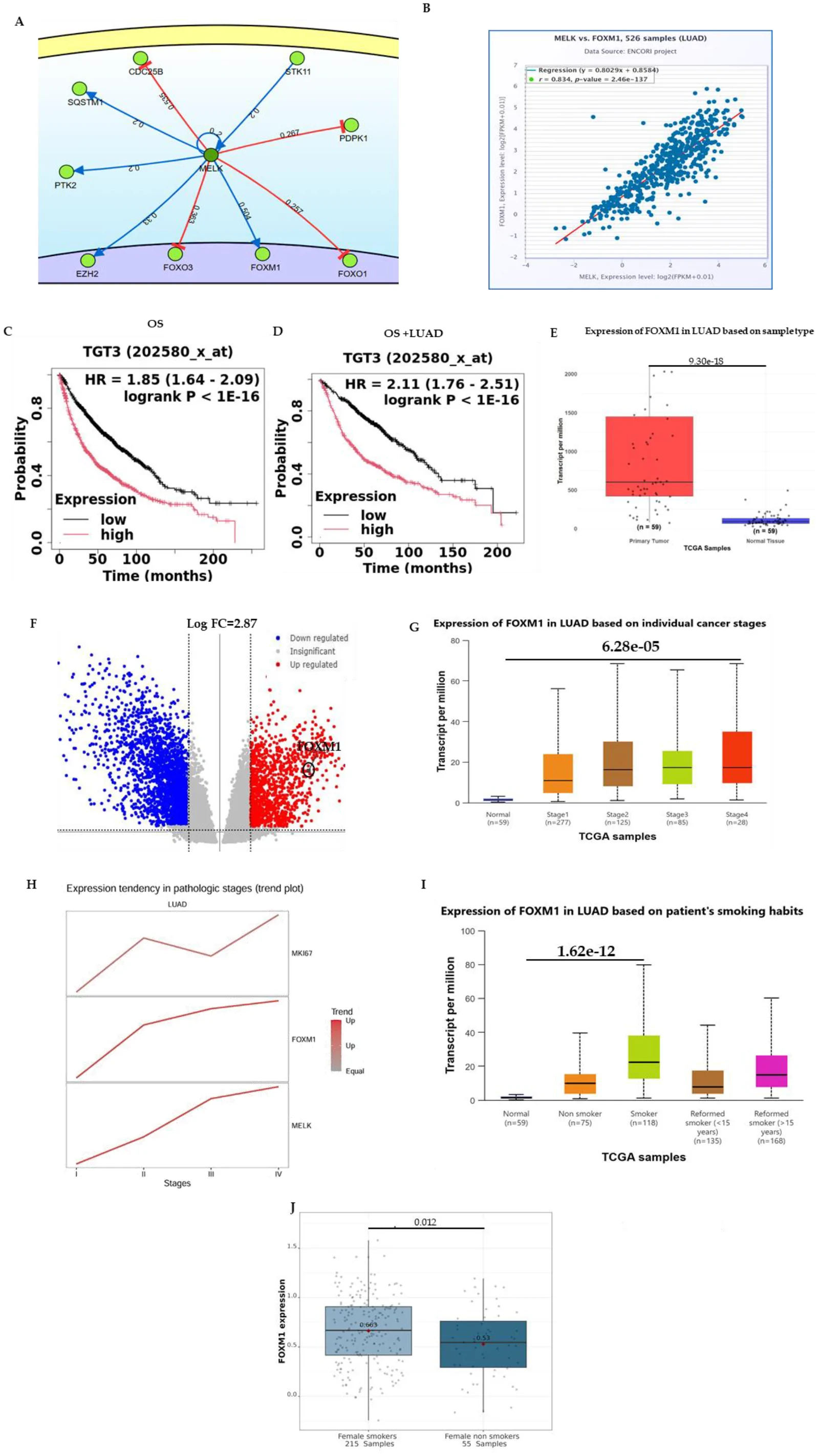
Expression and functional correlation of MELK and FOXM1 in lung adenocarcinoma. **(A)** SIGNOR database directed signaling model showing MELK regulation. **(B)** ENCORI showing correlation between MELK and FOXM1. KM Plotter analysis of FOXM1 in **(C)** OS (n=2166) and **(D)** OS+LUAD (n=1161). **(E)** FOXM1 expression in tumor vs. normal by using the R (normal n = 59, tumor n =59). **(F)** TCGA AnalyzeR showing FOXM1 transcriptome analysis. FOXM1 expression based on cancer stages by using **(G)** UALCAN and **(H)** GSCA. **(I)** FOXM1 expression based on patients’ smoking habits by using UALCAN and **(J)** FOXM1 expression in female smokers vs. non-smoker females by using LCE.

Cancer governs tumor heterogeneity by differentially dysregulating multiple genes and pathways, ultimately shaping tumor growth, evolution, and treatment response. In this context, a heterogeneous correlation model was constructed by analyzing potential regulators (positively and negatively associated) of MELK in LUAD. Initially, genes were identified using the UALCAN database, and their correlations with MELK expression were subsequently validated through the ENCORI platform (**Supplementary Table 6**). Among the top positively correlated genes, CKAP2L, KIF4A, NCAPG, CCNA2, CDCA5, MELK, BUB1, SKA1, CCNB1, and DEPDC1 showed strong associations (R ≥ 0.81), suggesting potential co-regulatory roles. Conversely, negatively correlated genes, including NAPSA, TMEM125, C17orf108, C16orf89, SELENBP1, RILP, LMF1, SLC22A3, SFTPB, and CD81 (R ≤ -0.41), showed distinct inverse expression patterns, suggesting divergent regulatory clusters within the MELK-associated gene network in LUAD. To further explore protein-protein and signaling associations, STRING analysis revealed a tightly connected interaction network between MELK and cell cycle-related genes, including FOXM1, CDC20, BUB1, CCNB1, PBK, etc., as illustrated in **Supplementary Figure 3H**. Additionally, we explored regulatory relationships by examining MELK-correlated genes within the SIGNOR signaling database. The SIGNOR-directed signaling model identified several candidate regulators, including FOXM1, SQSTM1, PTK2, EZH2, FOXO3, FOXO1, PDPK1, STK11, and CDC25B, as shown in **Figure 3A**. The correlations between MELK and these candidate genes were subsequently validated using independent datasets from ENCORI, OncoDB, and GEPIA2, with the corresponding correlation values summarized in **Supplementary Table 7**. Among all candidates, FOXM1 showed consistently high positive correlations with MELK across all databases (SIGNOR: R = 0.504; ENCORI: R = 0.834; OncoDB: R = 0.836; GEPIA2: R = 0.854), exceeding the predefined correlation cutoff of R > 0.5. In contrast, the remaining candidates exhibited weaker or negative correlations across datasets. Based on these observations, FOXM1 was selected for subsequent downstream analyses. FOXM1 is a key transcriptional activator of various G2/M phase molecules (50); its further association with MELK was visualized using the ENCORI database, and a strong positive correlation (R = 0.834), as shown in **Figure 3B**, underscored their co-regulatory relationship. Subsequently, survival analysis using the KMP database was performed to assess the prognostic impact of FOXM1 (TGT3) expression across various clinical parameters. Overall, elevated FOXM1 expression was significantly associated with poor OS (HR = 1.85, 95% CI = 1.64-2.09, P = <1e -16) (**Figure 3C**), FP (HR = 1.87, P = 3.6e-13), and PPS (HR = 1.42, P = 0.00095) in lung cancer patients (**Supplementary Table 8**). Subsequently, the multivariate Kaplan-Meier analysis also revealed that high FOXM1 expression was significantly associated with poor OS in LUAD patients (HR = 2.11, 95% CI = 1.76-2.51, P < 1e-16) (**Figure 3D****).** When stratified by gender, high FOXM1 expression remained an unfavorable prognostic factor in both males (HR = 1.83, 95% CI = 1.44–2.32, P = 4.8e-07) and females (HR = 2.35, 95% CI = 1.77–3.13, P = 1.3e-09). However, among female smokers, elevated FOXM1 expression was also associated with significantly reduced survival (HR = 2.08, 95% CI = 1.38-3.14, P = 0.00035) (**Supplementary Table 8**).

Further analysis of FOXM1 differential expression levels across multiple TCGA databases, including UALCAN and ENCORI, consistently showed significant upregulation of FOXM1 in LUAD tumor tissues compared to normal lung samples (**Supplementary Figures 3I, J**). Validation using R-based differential expression analysis with normalized TCGA samples (N = 59 each for tumor and normal, P = 9.30e-18), as shown in **Figure 3E**, confirmed a marked increase in FOXM1 expression in LUAD tumor samples. This was further supported by TCGAnalyzeR data, which reported a logFC of 2.87, confirming FOXM1 transcript overexpression in LUAD (**Figure 3F**). The UALCAN database further revealed that FOXM1 expression gradually increased with advancing cancer stages (I–IV) (p < 0.001), suggesting its potential role in disease progression, as shown in **Figure 3G**. Consistently, GSCA analysis revealed elevated expression of FOXM1 and MELK, along with the proliferation marker MKI67, across LUAD pathological stages, reflecting their coordinated involvement in tumor development (**Figure 3H**). Further, UALCAN analysis revealed that FOXM1 expression progressively increased with advancing nodal metastasis stages in LUAD (**Supplementary Figure 3K**) and was also significantly elevated in smoker groups compared with non-smokers (**Figure 3I**), suggesting that FOXM1 activation is linked to both metastatic progression and smoking status. Supporting this, LCE also showed elevated FOXM1 expression in female smokers compared with non-smokers (P = 0.012; **Figure 3J**), underscoring the influence of smoking on FOXM1 expression in LUAD. Accordingly, given MELK’s association with cell-cycle regulation and EMT, we further evaluated its predicted transcription factor, FOXM1, to determine its relationship with key G2/M cell-cycle regulators and EMT-associated markers. FOXM1 expression exhibited strong positive correlations with key G2/M cell-cycle regulators, including CDK1 (R = 0.775), CCNB1 (R = 0.795), PLK1 (R = 0.891), and CDC25C (R = 0.791) by ENCORI; these correlations support the involvement of FOXM1 in G2/M phase regulation (**Supplementary Table 18**). Consistent with the association observed between MELK and EMT markers, FOXM1 displayed a moderate positive correlation with the EMT regulators TWIST1 (R = 0.307) and SNAI1 (R = 0.383) (**Supplementary Table 19**). These findings highlight the FOXM1-MELK axis as a potential driver of LUAD progression and a promising prognostic indicator. Based on integrated bioinformatics and correlation analyses, we proposed that FOXM1 overexpression in LUAD may transcriptionally regulate MELK, leading to increased MELK mRNA levels and overexpression in tumor tissues, as shown in **Supplementary Figure 3G**.

### 3.6 MELK-associated tumor immune microenvironment

**Figure 4:**
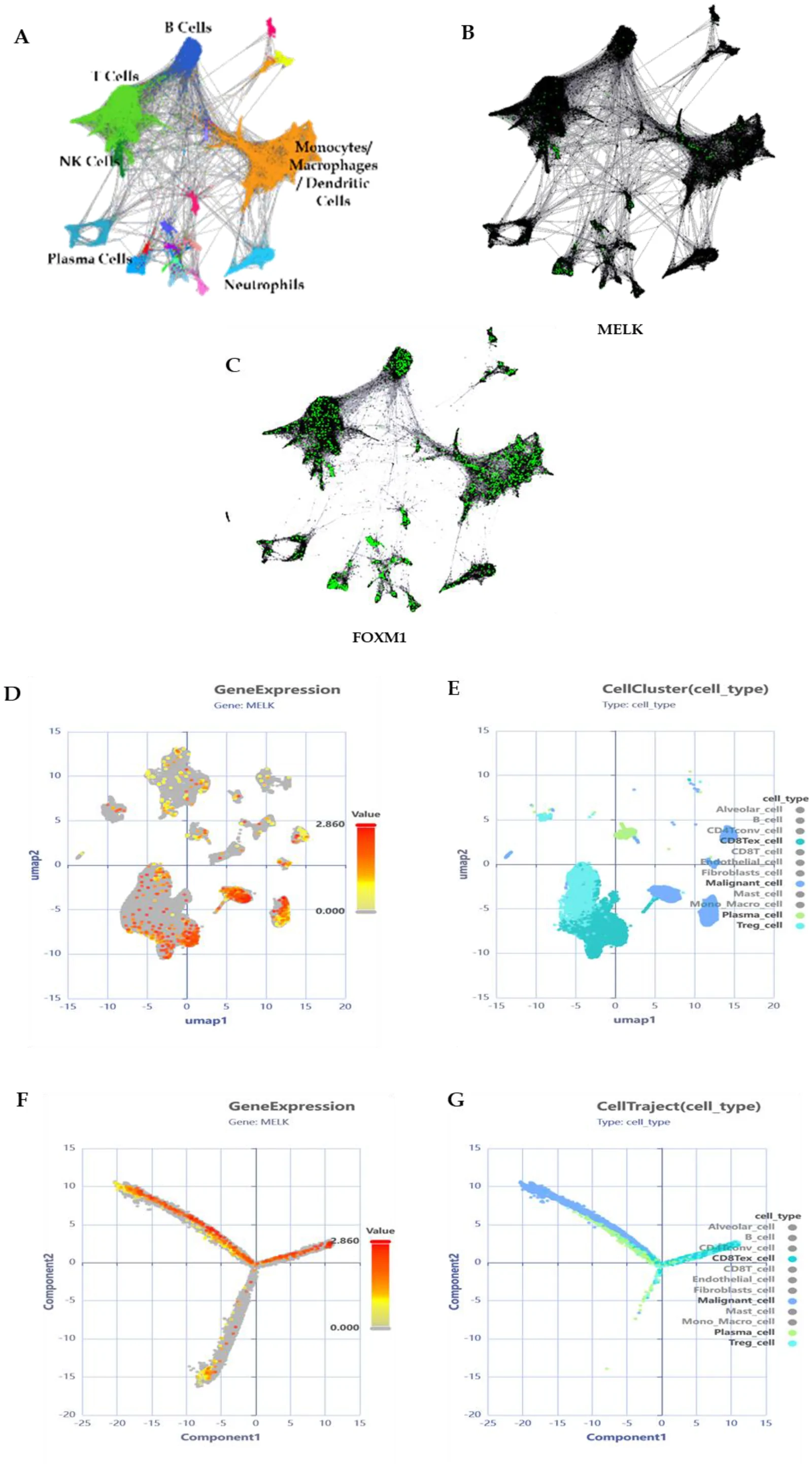
Single-cell analysis of MELK and FOXM1 in NSCLC immune microenvironment. **(A–C)** Spring viewer immune cell expression maps showing MELK and FOXM1 distribution across the immune cell population. **(D)** UMAP plots showing MELK gene expression. **(E)** MELK gene expression corresponding to immune cell clusters. **(F)** Pseudotime trajectory showing MELK gene expression. **(G)** associated cell type distribution.

Given the critical role of TIME in determining the success of immunotherapies, we analyzed MELK and its transcriptional factor, FOXM1, to examine their association with immune infiltration in NSCLC. Using the Spring viewer, we observed distinct MELK and FOXM1 expression across major immune cell populations, including T cells, B cells, macrophages, dendritic cells, NK cells, neutrophils, and plasma cells, as shown in **Figures 4A–C**. Each of these plays a unique role: T cells mediate cytotoxic (CD8^+^) and helper (CD4^+^) immune functions; B cells produce antibodies and present antigens; macrophages and dendritic cells regulate antigen presentation and immune signaling; NK cells contribute to direct tumor cell lysis; neutrophils drive inflammatory responses; and plasma cells support humoral immunity (54, 55). Since Spring Viewer provides a qualitative overview, we further validated the predictive relevance of these genes using ROC curve analysis in TIMER 3.0. Both MELK (AUC = 0.693) and FOXM1 (AUC = 0.689) showed moderate predictive value for immune responsiveness, suggesting their potential role in immune regulation and evasion mechanisms within NSCLC (**Supplementary Figures 4A, B**).

Following the immune expression mapping, we evaluated the association between MELK expression and various immune cell populations using TIMER2.0 to identify specific immune subsets influenced by MELK activity. Among the heterogeneous immune populations, MELK showed significant negative correlations with CD8^+^ T cells (R = -0.231), CD4^+^ T cells (R = -0.334), B cells (R = -0.337), macrophages (R = -0.39), and dendritic cells (R = -0.327), while displaying moderate links with neutrophils and monocytes. To further refine the immune interaction profile, we compared MELK with its positively and negatively correlated cell cycle regulators across the TIME (**Supplementary Table 9****),** thereby identifying immune subsets that consistently aligned with MELK expression. The immune cell types, including hematopoietic stem cells (HSCs), myeloid derived suppressor cells (MDSCs), macrophages (M2), dendritic cells, cancer associated fibroblasts (CAFs), and B cells, emerged as key immune partners associated with the MELK regulatory axis. These correlations suggest that MELK may play an active role in shaping the TIME, potentially supporting immune suppression and tumor promoting inflammation through its association with myeloid and stromal compartments, while still maintaining partial interactions with adaptive immune cells such as T and B lymphocytes. Additionally, the GSCA analysis revealed a consistent alteration in T-cell associated immune populations in LUAD. Notably, CD4^+^ T-cell infiltration showed the strongest negative association across all analyzed genes, including MELK, FOXM1, TWIST1, SNAI1, CCNB1, CDK1, and TMPO-AS1, indicating a pronounced depletion of CD4^+^ T cells in tumors with high expression of these molecules. In contrast, regulatory T cells (Tregs) exhibited a strong positive correlation with elevated expression of all assessed genes, reflecting a coordinated enrichment of immunosuppressive T-cell populations (**Supplementary Figure 4C**).

To further characterize MELK expression at the single-cell level, we used the CellTracer platform and selected the NSCLC_EMTAB6149 dataset, which profiles tumor and immune cell populations in NSCLC. We first examined the class-based clusters (**Supplementary Figure 5A**) and then visualized the immune cell-specific clusters (**Supplementary Figure 5B**) using UMAP projection, which revealed distinct groupings of immune and malignant cell types. Next, we queried the MELK gene and visualized its expression distribution across clusters, as shown in **Figure 4D**. We quantified mean expression per cell type (**Supplementary Figure 5C**) and applied a cut-off threshold of 0.02 to identify cell populations with relatively higher MELK expression. Based on this criterion, four major cell types, CD8^+^ exhausted T cells, malignant tumor cells, plasma cells, and regulatory T (Treg) cells, exhibited elevated MELK expression levels. We then refined the visualization, as shown in **Figure 4E**, to focus exclusively on these four enriched cell populations. The results highlighted a preferential enrichment of MELK within exhausted T cells and malignant compartments, suggesting that MELK may participate in immune exhaustion and tumor immune evasion while also being detectable in plasma and Treg cells that support immunosuppressive signaling in the TME.

Furthermore, we explored the developmental relationship among the MELK expressing immune and malignant cell populations, and we performed cell trajectory analysis using Monocle 2. The CellTraject (Cluster) plot (**Supplementary Figure 5D**) visualized the global pseudotime distribution across identified clusters, followed by the CellTraject (Cell Type) map (**Supplementary Figure 5E**), which annotated specific immune and tumor cell types along the trajectory. Subsequently, MELK expression was projected along the pseudotime curve (**Figure 4F**), revealing a distinct gradient pattern. Notably, CD8^+^ exhausted T cells, malignant tumor cells, plasma cells, and regulatory T cells (Tregs) showed elevated MELK expression throughout the developmental pathway, consistent with our single-cell clustering and expression analyses (**Figure 4G**). This trajectory-based validation further supports the involvement of MELK in tumor associated immune modulation and immune exhaustion, highlighting its potential functional role during NSCLC immune evolution.

### 3.7 Potential MELK therapeutic targets

Given the strong transcriptional, proteomic, and functional evidence supporting MELK as an oncogenic driver in LUAD, we comprehensively evaluated the ligandability of MELK and determined whether clinically relevant mitotic and checkpoint directed drugs can effectively target this kinase. We compared a broad panel of selective MELK inhibitors, G2/M checkpoint-targeting drugs, and bioactive natural compounds for MELK-binding potential. Using the well-established MELK inhibitor OTSSP167 (binding affinity; -9.672 kcal/mol) enabled benchmarking of binding strength and interaction reliability across all tested molecules. The results revealed that, in addition to the selective inhibitor, which exhibited strong and stable binding within the MELK catalytic pocket, several non-canonical ligands also demonstrated comparably high binding affinities, indicating pronounced structural compatibility with MELK. Notably, multiple G2/M checkpoint-targeting inhibitors demonstrated strong binding interactions within the MELK active site, suggesting functional overlap between MELK regulation and mitotic checkpoint control. The direct MELK inhibitor OTSSP167 showed high binding affinity (-9.672 kcal/mol), validating the docking approach. Among G2/M specific chemotherapeutic and checkpoint inhibitors, Onvansertib (-9.374 kcal/mol), Volasertib (-9.264 kcal/mol), Adavosertib (-9.296 kcal/mol), Alisertib (-8.993 kcal/mol), Barasertib (-8.906 kcal/mol), AZD7762 (-8.436 kcal/mol), RP-6306 (-8.070 kcal/mol), UCN02 (-8.057 kcal/mol), and Prexasertib (-7.260 kcal/mol) exhibited favorable engagement with the MELK binding pocket. Conventional microtubule targeting agents, including Paclitaxel (-7.753 kcal/mol), Docetaxel (-7.549 kcal/mol), Vinblastine (-7.642 kcal/mol), Vincristine (-6.930 kcal/mol), and Nocodazole (-7.247 kcal/mol), also showed moderate binding affinity, indicating potential indirect modulation of MELK- associated mitotic processes.

**Figure 5:**
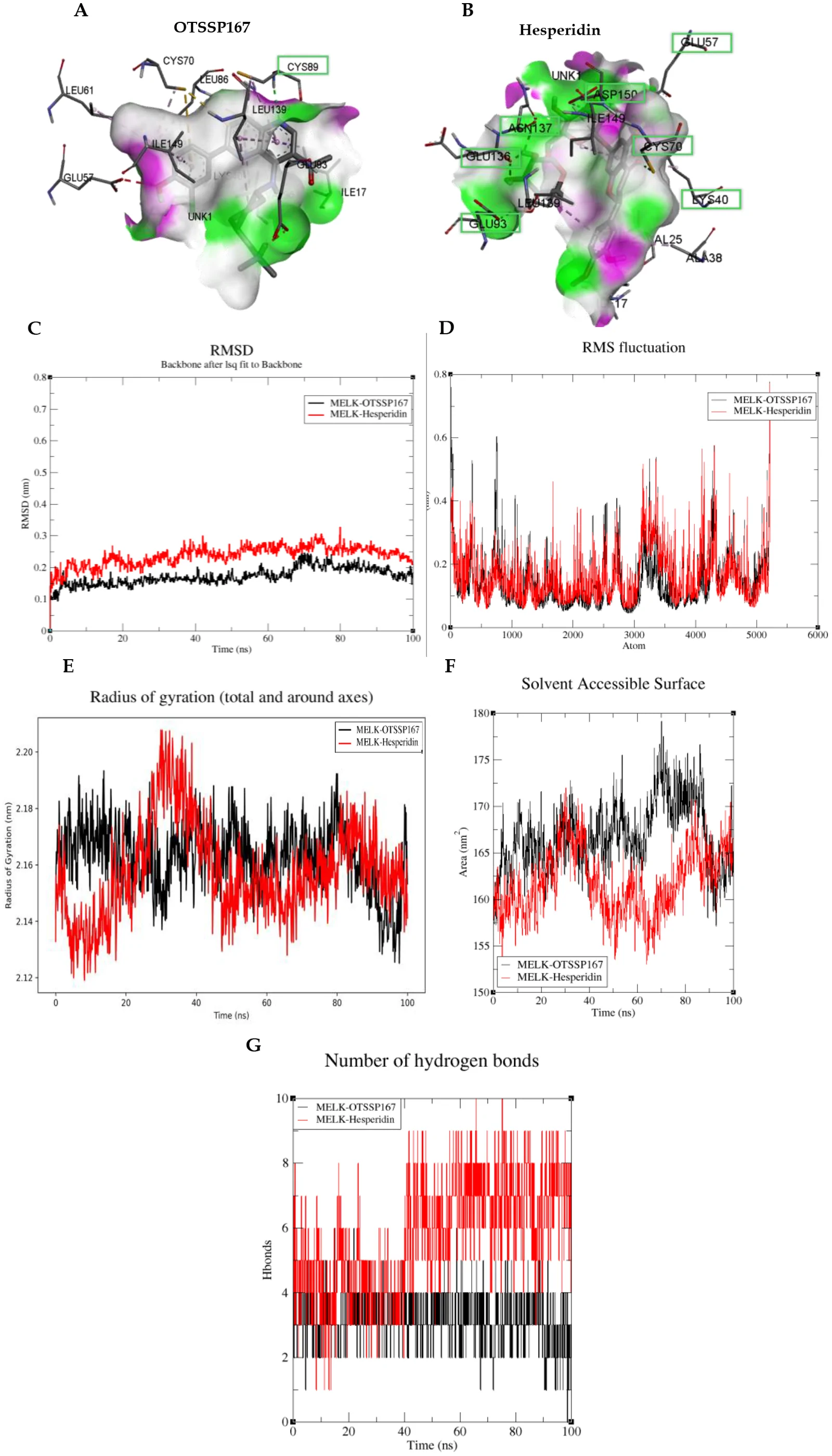
Docking interactions and molecular dynamics profiling of MELK–ligand complexes. 3D Binding models of **(A)** OTSSP167 and **(B)** Hesperidin within the MELK catalytic pocket, **(C)** Backbone RMSD trajectories over 100 ns indicating stable complex formation, with MELK-OTSSP167 and MELK-Hesperidin, **(D)** RMSF analysis showing residue-level flexibility, **(E)** Radius of gyration (Rg) demonstrating maintained structural compactness throughout the simulation, **(F)** Solvent-accessible surface area (SASA) reflecting moderate conformational dynamics upon ligand binding, **(G)** Hydrogen bond analysis.

Furthermore, natural flavonoid compounds demonstrated remarkable binding stability toward MELK. Hesperidin (-10.050 kcal/mol) emerged as the strongest natural binder, followed closely by Eriocitrin (-10.030 kcal/mol), Narirutin (-9.866 kcal/mol), Naringin (-9.556 kcal/mol), Neohesperidin (-9.556 kcal/mol), and Rutin (-9.361 kcal/mol). Additional flavonoids, including Fisetin (-8.545 kcal/mol), Quercetin (-8.411 kcal/mol), Hesperetin (-8.370 kcal/mol), Kaempferol (-8.367 kcal/mol), Isorhamnetin (-8.339 kcal/mol), and Myricetin (-8.214 kcal/mol), also exhibited binding affinities comparable to or exceeding several selective kinase inhibitors. All predicted binding affinities are summarized in **Supplementary Table 10**. Notably, Hesperidin emerged as the lead natural compound based on its superior binding affinity and favorable interaction profile with MELK. Collectively, these findings suggest that both synthetic checkpoint inhibitors and naturally occurring flavonoids may modulate MELK activity, highlighting MELK as a promising therapeutic target for cell cycle–based intervention strategies. The 2D interaction visualizations (**Supplementary Figures 6A–I**, **7A–H**, **8A–K**) confirmed these interactions, highlighting the consistent involvement of critical residues within the MELK active pocket across compounds. The 3D binding surface visualizations, as shown in **Figures 5A, B**, **Supplementary Figures 9A–H**, **10A–G**, **11A–K**, were incorporated, which clearly demonstrated compact ligand accommodation within the ATP-binding cleft and consistent pocket occupation patterns across all ligand classes, further supporting the stability and accuracy of the predicted docking poses. Collectively, these findings demonstrate that MELK contains a structurally permissive and functionally adaptable ligand binding-cavity capable of accommodating a broad spectrum of chemically diverse therapeutic scaffolds. Importantly, beyond selective MELK inhibitors, both G2/M checkpoint-targeting agents and naturally derived flavonoids may exert additional anticancer effects by directly engaging or modulating MELK. This suggests that MELK may serve as a convergent regulatory node linking mitotic control and therapeutic response, thereby providing a strong rationale for drug repurposing and the rational design of combination based therapeutic strategies in cancer.

### 3.8 Molecular dynamics simulation confirms stable MELK-ligand complex formation

To evaluate whether the favorable docking interactions were maintained under dynamic conditions, comparative MD simulations were performed for the MELK-OTSSP167 and MELK-Hesperidin complexes. The quantitative summary of the molecular dynamics simulation parameters is presented in **Supplementary Table 11**. Both complexes remained structurally stable throughout the 100 ns simulation, with RMSD profiles reaching equilibrium after the initial equilibration phase and exhibiting no significant structural drift (**Figure 5C**). Although the MELK-Hesperidin complex showed slightly higher RMSD values than the reference inhibitor, the observed fluctuations remained within an acceptable range of 1 amstrong, indicating stable binding throughout the simulation. RMSF analysis demonstrated comparable residue flexibility in both complexes, with increased fluctuations primarily restricted to flexible loop regions (**Figure 5D**). Likewise, the radius of gyration and SASA remained relatively constant during the simulation, indicating preservation of the overall structural compactness and solvent accessibility of MELK in both ligand-bound states (**Figures 5E,F**). Furthermore, stable hydrogen-bonding profiles were maintained throughout the simulation, supporting the structural integrity of both systems (**Figure 5G**). Collectively, these findings demonstrate that Hesperidin maintains stable association with MELK under dynamic physiological conditions, exhibiting structural behavior comparable to the clinically established inhibitor OTSSP167. Although OTSSP167 displayed slightly greater conformational stability, the sustained binding and preserved structural integrity observed for Hesperidin provide additional computational support for its potential use as a natural agent in combination with MELK-targeted therapies, rather than as a monotherapy.

### 3.9 Identification and selection of regulatory miRNAs associated with MELK and FOXM1 in LUAD

To explore the post-transcriptional regulation of the MELK-FOXM1 axis in LUAD, 31 candidate miRNAs predicted to co-target both MELK and FOXM1 were initially identified using the miRNet database (**Supplementary Figure 12B**). The expression patterns of all 31 miRNAs were first assessed using the UALCAN database. Among them, seven miRNAs, hsa-let-7b-5p, hsa-miR-98-5p, hsa-let-7g-5p, hsa-miR-29c-3p, hsa-miR-221-3p, hsa-miR-138-5p, and hsa-miR-126-3p, were found to be significantly downregulated in LUAD tissues compared to normal controls (**Supplementary Table 12****).** Correlation analysis was performed of these 7 downregulated miRNAs through ENCORI (**Supplementary Table 13**) with MELK and FOXM1. Among them, the downregulated miRNAs, namely hsa-let-7b-5p (R = -0.360 with MELK; R = -0.393 with FOXM1), hsa-miR-29c-3p (R = -0.393 with MELK; R = -0.406 with FOXM1), hsa-let-7g-5p (R = -0.171 with MELK; R = -0.191 with FOXM1), and hsa-miR-126-3p (R = -0.078 with MELK; R = -0.123 with FOXM1), showed negative correlations, suggesting their possible tumor-suppressive role in LUAD. In contrast, hsa-miR-98-5p and hsa-miR-138-5p displayed weak or positive associations, indicating a limited regulatory effect. Among these, hsa-let-7b-5p and hsa-miR-29c-3p were prioritized for further analysis based on their strong negative correlations and consistent downregulation across databases (**Supplementary Figures 12C–F**). Survival analysis using the KMP database revealed that elevated expression of hsa-let-7b-5p (HR = 0.72, P = 0.026) and hsa-miR-29c-3p (HR = 0.54, P = 0.0012) was significantly associated with improved overall survivability in LUAD patients (**Supplementary Figures 12G, H**). To validate these findings, the CancermiRNome database was used to examine their prognostic impact using ROC analysis, where hsa-let-7b-5p exhibited a high diagnostic potential (AUC = 0.89; 95% CI: 0.85-0.92) as compared to hsa-mir-29c-3p (AUC = 0.67, 95% CI: 0.58-0.77) (**Supplementary Figures 12I, J**). Based on this observation, we decided to further examine all the members of the let-7 family using KMP and ROC analysis. Interestingly, out of 10 let-7 family members (**Supplementary Table 14**), hsa-let-7b-5p, hsa-let-7c-5p, and hsa-miR-202 were found to be significant tumor suppressors. Further, AUC analysis using CancermiRNome revealed hsa-let-7b-5p and hsa-let-7c-5p to be regulatory miRNA candidates (**Supplementary Figures 12K, L**). Additionally, binding analysis using miRWalk and RNA22v2 confirmed a stronger predicted target site for hsa-let-7b-5p within the MELK transcript as compared to hsa-let-7c-5p. Let-7b-5p binds to MELK at position 1093, with a favorable binding energy of -20.1 and folding energy of -16.7 kcal/mol, indicating a stable interaction (**Supplementary Table 15****).**

**Figure 6:**
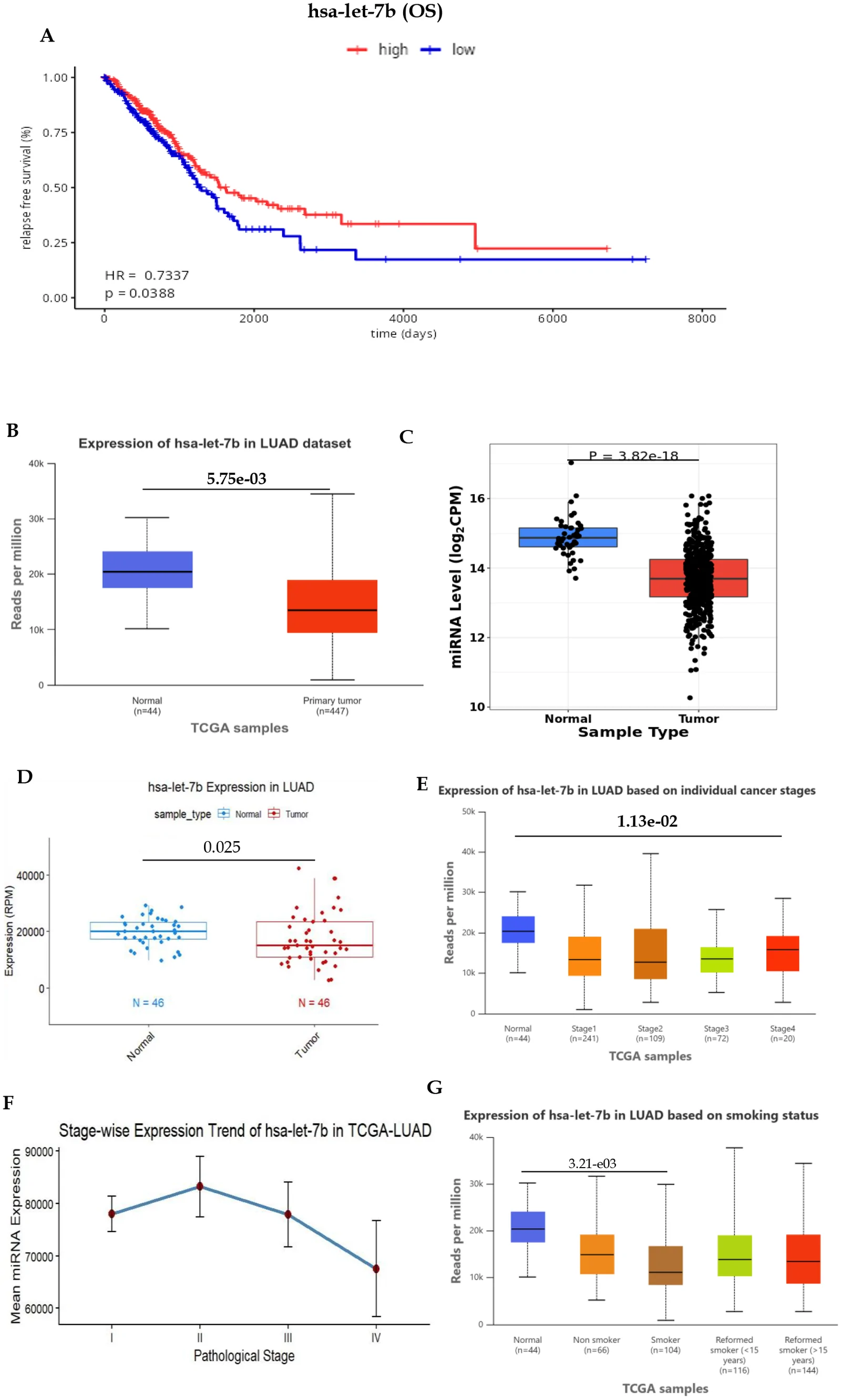
Expression and prognostic significance of hsa-let-7b in lung adenocarcinoma (LUAD). **(A)** Overall survival plot of hsa-let-7b-5p by using UALCAN (miRTarget). Expression of hsa-let-7b-5p in LUAD tumors vs. normal tissues by using **(B)** UALCAN (normal n = 44, tumor n = 447), **(C)** CancerMIRNome and **(D)** R (normal n = 46, tumor n =46). Stage wise hsa-let-7b-5p expression trend **(E)** UALCAN **(F)** an R-based package. **(G)** Expression of hsa-let-7b-5p based on smoking status.

As illustrated in **Figure 6A**, Kaplan-Meier survival analysis using UALCAN (miR target) showed that low hsa-let-7b-5p expression was significantly associated with poorer OS in LUAD patients (HR = 0.7337, P = 0.0388), suggesting a potential prognostic role. In **Figure 6B**, also derived from UALCAN, hsa-let-7b-5p expression was significantly downregulated in LUAD tumor tissues compared to normal samples (P = 5.75e-03). This observation was further supported by **Figure 6C**, which showed that cancermiRNome analysis similarly demonstrated reduced levels of hsa-let-7b-5p in tumor tissues (P = 3.82e-18), confirming its consistent downregulation across datasets. Additionally, **Figure 6D**, generated using R and normalized sample data (n = 46), served as an internal validation and confirmed the differential expression pattern of hsa-let-7b-5p between normal and LUAD tissues (P = 0.025). Clinicopathological analysis using UALCAN revealed significantly higher hsa-let-7b-5p expression in normal lung tissues compared with tumor samples across different pathological stages (**Figure 6E****).** Consistently, R-based stagewise trend analysis demonstrated a gradual decline in hsa-let-7b-5p expression with advancing pathological stages of LUAD (**Figure 6F**). In addition, UALCAN analysis showed elevated hsa-let-7b-5p levels in normal lung tissues compared with samples from smokers and those with nodal metastasis (**Figure 6G****;** **Supplementary Figure 12M**). Accordingly, based on the observed involvement of MELK and its transcription factor FOXM1 in cell-cycle regulation and EMT, we next evaluated the association of the related miRNA hsa-let-7b-5p with key G2/M cell-cycle regulators and EMT-associated markers. We analyzed its correlation with key G2/M regulators; hsa-let-7b-5p expression exhibited moderate negative correlations with key G2/M cell-cycle regulators, including CDK1 (R = -0.319), CCNB1 (R = -0.378), PLK1 (R = -0.382), and CDC25C (R = -0.382). This correlational pattern suggests that hsa-let-7b-5p expression is inversely linked to G2/M cell-cycle regulatory activity (**Supplementary Table 19**). Further, correlation analysis revealed that hsa-let-7b-5p expression was inversely associated with the EMT regulators TWIST1 (R = -0.062) and SNAI1 (R = -0.148) (**Supplementary Table 20**). Although the correlation values were weak, the observed inverse association suggests that hsa-let-7b-5p may contribute to the suppression of EMT marker expression and potentially inhibit EMT progression. Based on these observations, a working model was proposed in which FOXM1-mediated transcriptional activation, together with reduced hsa-let-7b-5p expression, contributes to elevated MELK expression in LUAD (**Supplementary Figure 12A**).

### 3.10 Identification of hsa-let-7b-5p associated lncRNAs

**Figure 7:**
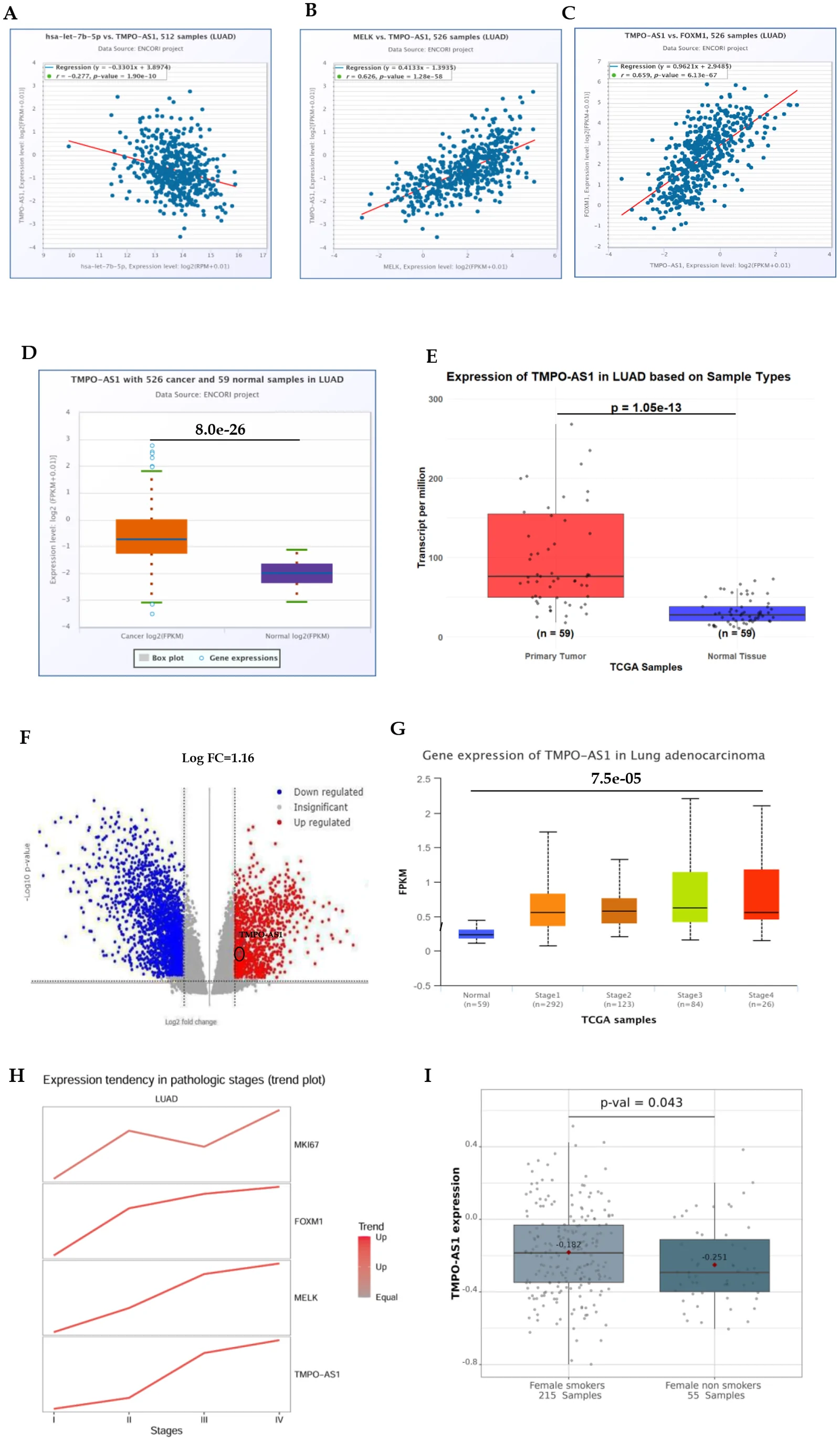
Correlation and clinical expression analysis of TMPO-AS1 in lung adenocarcinoma (LUAD). Correlation analysis of TMPO-AS1 with **(A)** hsa-let-7b-5p, **(B)** MELK, and **(C)** FOXM1 by using ENCORI. Expression profiling of TMPO-AS1 in LUAD tumors vs. normal using **(D)** ENCORI (normal n = 59, tumor n = 526) and **(E)** R (normal n = 59, tumor n =59). **(F)** TMPO-AS1 transcriptome analysis by using TCGAnalyzeR. TMPO-AS1 expression based on cancer stage by using **(G)** UALCAN and **(H)** GSCA. **(I)** LCE comparative analysis of TMPO-AS1 expression in female smokers vs. non-smoker females.

To elucidate the upstream regulatory network of hsa-let-7b-5p, we first identified its potential interacting lncRNAs using the Enrichr database (**Supplementary Table 16****).** Among the 97 predicted candidates, 37 lncRNAs showed significantly elevated expression levels in LUAD based on UALCAN analysis. Out of these, VPS9D1-AS1, DDX11-AS1, TMPO-AS1, and ELFN1-AS1 showed significant associations with hsa-let-7b-5p, MELK, and FOXM1, analyzed using ENCORI (**Supplementary Table 17**). To validate the prognostic value of these lncRNAs, survival analysis was performed using the KMP database. Among all the lncRNAs, only TMPO-AS1 displayed a significant HR = 1.50, 95% CI = 1.29-1.74, P = 7.2e-08, for OS in lung cancer patients, with a particularly strong prognostic impact in LUAD cohorts (HR = 2.16, P = 4.1e-10), especially among female smokers (HR = 3.13, P = 0.016) (**Supplementary Table 18**). Additionally, pan-cancer expression analysis also revealed that TMPO-AS1 was significantly upregulated in tumor tissues compared with normal tissues across multiple cancer types in the TCGA cohort. Among these malignancies, LUAD also demonstrated a significant increase in TMPO-AS1 expression relative to normal lung tissue, supporting its potential involvement in lung adenocarcinoma progression, as shown in **Supplementary Figure 13A**. Further, to evaluate the expression and regulatory significance of TMPO-AS1 in LUAD, correlation analyses from the ENCORI database revealed a significant negative association between hsa-let-7b-5p and TMPO-AS1 expression in LUAD samples (R = -0.217, P = 1.20e-05) as shown in **Figure 7A**, suggesting that TMPO-AS1 may function as a ceRNA by sponging hsa-let-7b-5p. The miRNA hsa-let-7b-5p sequences were then derived from miRWalk, and RNA22v2 analyses revealed a stable predicted binding interaction between TMPO-AS1 and hsa-let-7b-5p, supported by favorable folding energy (-13.90 kcal/mol) and a well-aligned heteroduplex structure, as mentioned in **Table 2**. In contrast, a strong positive correlation was observed between MELK and TMPO-AS1 (R = 0.601, P = 1.28e-59), as well as between FOXM1 and TMPO-AS1 (R = 0.513, P = 1.64e-38), indicating their potential co-expression and role in LUAD proliferation pathways, as shown in **Figures 7B, C**, respectively. Further, data from ENCORI, OncoDB, UALCAN, and LCE confirmed a significant overexpression pattern of TMPO-AS1 in LUAD samples (**Figure 7D****;** **Supplementary Figures 13B–D**). Validation using R-based differential expression analysis with normalized TCGA samples (n = 59 each for tumor and normal, P = 1.05e-13), as shown in **Figure 7E**, confirmed a marked increase in TMPO-AS1 expression in LUAD tumor samples. This was further supported by data from TCGAnalyzeR, which reported a logFC of 1.16, confirming the overexpression of TMPO-AS1 transcripts in LUAD, as illustrated in **Figure 7F**. Furthermore, UALCAN stage-wise analysis showed a gradual increase in TMPO-AS1 expression from stage I to stage IV, suggesting its involvement in disease progression (**Figure 7G**). Consistently, GSCA analysis confirmed a rising expression trend of TMPO-AS1 with MELK, FOXM1, and MKI67 across LUAD pathological stages, reflecting their coordinated involvement in tumor development, as shown in **Figure 7H**. Moreover, LCE identified a significant difference in TMPO-AS1 expression between female smokers and non-smokers (P = 0.043), as shown in **Figure 7I**. Furthermore, within the MELK-centered ceRNA regulatory framework linking MELK and FOXM1, we evaluated the association of the lncRNA TMPO-AS1 with key G2/M cell-cycle regulators and EMT-associated markers. We examined its expression correlations with key G2/M regulators. TMPO-AS1 showed a positive correlation with key G2/M cell-cycle regulators, including CDK1 (R = 0.626), CCNB1 (R = 0.581), PLK1 (R = 0.638), and CDC25C (R = 0.622). These findings support a positive association between TMPO-AS1 and a key G2/M regulator that promotes cell cycle progression (**Supplementary Table 19**). Given its association with G2/M regulators, we further evaluated the relationship between TMPO-AS1 and EMT-associated markers. TMPO-AS1 exhibited modest positive correlations with the EMT-associated markers TWIST1 (R = 0.230) and SNAI1 (R = 0.172), indicating a potential association with epithelial-mesenchymal transition (**Supplementary Table 20**).

**Table 2:** Binding affinity of hsa with TMPO-AS1.

miRNA name	Transcript name	Folding energy (RNA22v2) (kcal/mol)	Heteroduplex
hsa-let-7b-5p	TMPO-AS1 NM_001445508.1	-13.90	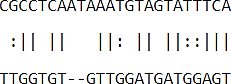

To further examine potential regulatory interactions, a miRNA-lncRNA interaction network was constructed using miRNet (**Supplementary Figure 13E**), which revealed that TMPO-AS1 and MELK are interconnected via hsa-let-7b-5p, suggesting a potential lncRNA-miRNA-mRNA regulatory relationship. Furthermore, the mechanistic model in Cytoscape (**Supplementary Figure 13F**) illustrates the core TMPO-AS1/hsa-let-7b-5p/MELK-FOXM1 axis, showing how TMPO-AS1 may sponge hsa-let-7b-5p, thereby reducing its suppressive effect on MELK and FOXM1. This reduction in miRNA-mediated inhibition may subsequently elevate MELK and FOXM1 expression, promoting proliferative signaling in LUAD. These findings support TMPO-AS1 as a key oncogenic lncRNA that promotes LUAD progression by modulating the hsa-let-7b-5p/MELK/FOXM1 regulatory axis, highlighting its potential as a biomarker and therapeutic target in LUAD. Hence, reestablishing the regulatory axis targeting the small molecule, such as miRNA hsa-let-7b-5p and lncRNA TMPO-AS1, could be an efficient prognostic target.

### 3.11 Expression profiling of TMPO-AS1/hsa-let-7b-5p/MELK axis in cell line model

**Figure 8:**
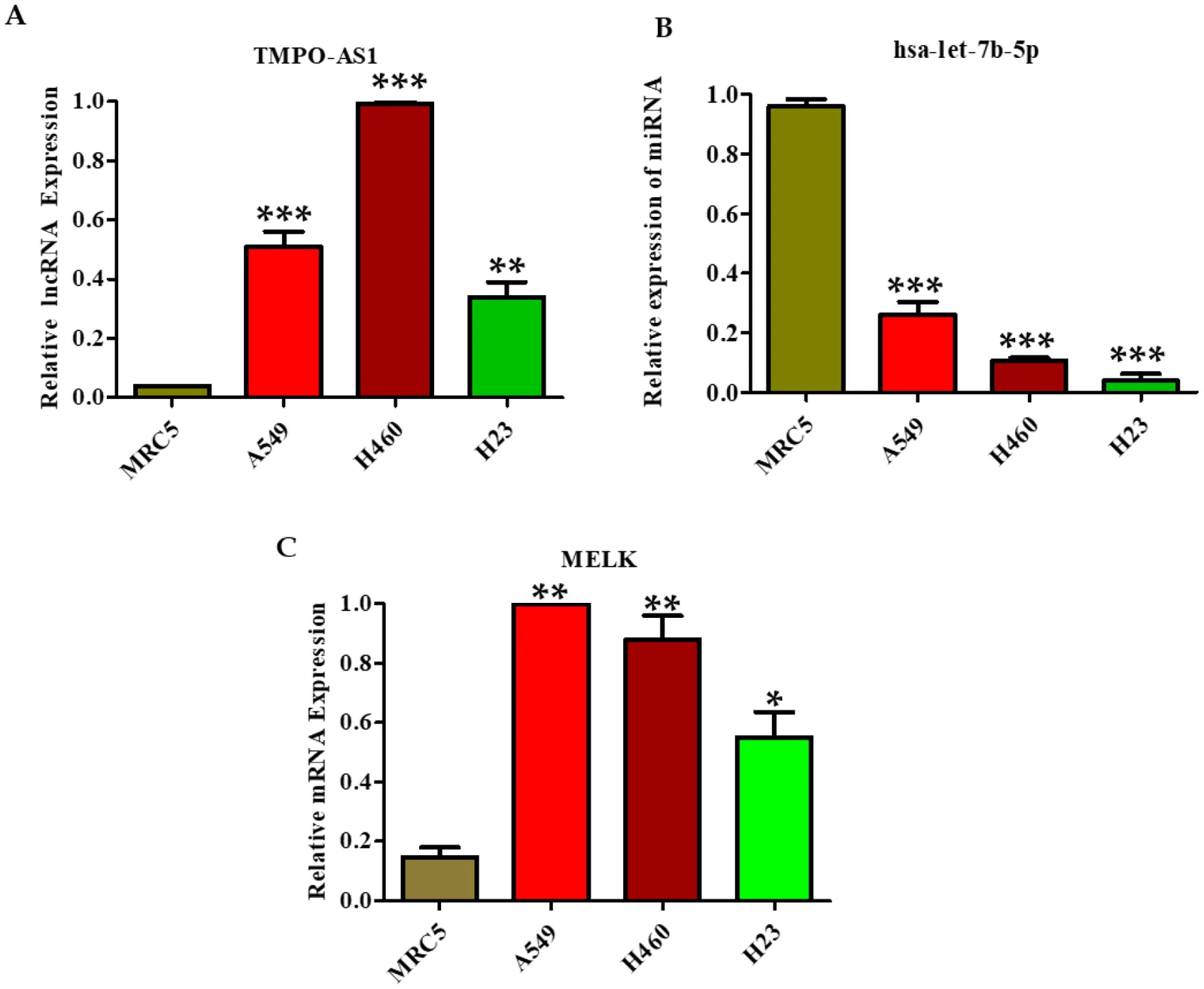
Expression profiling of TMPO-AS1, hsa-let-7b-5p, and MELK in lung cancer cell line model. **(A)** Relative expression of TMPO-AS1 in lung cancer cell lines (A549, H460, and H23) compared with the normal lung fibroblast cell line (MRC-5, used as control). **(B)** Relative expression of hsa-let-7b-5p in A549, H460, and H23 cells compared with MRC-5 control cells. **(C)** Relative mRNA expression of MELK in A549, H460, and H23 cells compared with MRC-5 control cells. ***p < 0.0001, **p < 0.001, *p < 0.05.

To validate the in silico findings, we performed experimental expression profiling of the TMPO-AS1/hsa-let-7b-5p/MELK axis in lung cancer cell line models. The relative expression of lncRNA TMPO-AS1 was experimentally validated in normal lung cells (MRC-5) and lung cancer cell lines (A549, H460, and H23) using qRT-PCR analysis. TMPO-AS1 expression was markedly upregulated in all lung cancer cell lines compared with MRC-5 cells, showing an approximate >20-fold increase in H460 cells, ~10-15-fold in A549 cells, and ~4-8-fold in H23 cells (**Figure 8A**). Furthermore, hsa-let-7b-5p expression was assessed in these cell lines (**Figure 8B**), and the results showed that hsa-let-7b-5p was significantly downregulated in all lung cancer cell lines compared with normal cells. Among them, H23 cells exhibited the highest downregulation, showing approximately a 10-fold change compared to MRC5, followed by H460 and A549 cells, suggesting a potential tumor-suppressive role of hsa-let-7b-5p in lung cancer. In addition, MELK expression was analyzed, revealing significant upregulation in all lung cancer cell lines. MELK expression showed an approximate fold change of ~4–5 in A549 cells, ~4–5 in H460 cells, and ~3–4 in H23 cells relative to MRC-5, with the highest expression observed in A549 cells (**Figure 8C**). The elevated expression of TMPO-AS1, along with the upregulation of MELK and downregulation of hsa-let-7b-5p, supports the proposed regulatory relationship among these molecules.

## 4 Discussion

LUAD is a common and lethal malignancy often diagnosed late, leading to poor outcomes (56). It is hindered by tumor heterogeneity, immune evasion, and treatment resistance. Outcomes vary by geography; for example, Asian patients have higher EGFR mutation rates, compared to Western cohorts, which affect treatment responses (57). LUAD exhibits chromosomal instability that drives evolution, therapy resistance, and metastasis, with mitotic regulators such as FOXM1, PLK1, and AURKA promoting unchecked division. MELK is a key driver of LUAD proliferation, supporting G2/M progression by regulating the PLK1-CDC25C-CDK1 axis (11). MELK is elevated in many cancers and is associated with poor survival outcomes (49–51). In LUAD, high MELK expression may serve as a prognostic biomarker and therapeutic target. Furthermore, FOXM1, a central mitotic transcription factor, promotes G2/M progression and genomic instability (58). Our study further identifies a strong association between FOXM1 and MELK in LUAD, particularly in late-stage and smoker-associated tumors, extending previous observations by highlighting the MELK–FOXM1 relationship within the LUAD-specific context. Although immune checkpoint inhibitors have transformed LUAD therapy, only a subset of patients derive durable benefit. The interplay between MELK and the immune microenvironment adds another dimension to its oncogenic relevance.

A further finding of our study was the association of high MELK expression with reduced infiltration of effector immune cells, CD8^+^ T cells, CD4^+^ T cells, B cells, macrophages, dendritic cells, and natural killer cells and preferential enrichment in exhausted CD8^+^ T cells and Tregs. By contrast, hsa-let-7b-5p correlates with more CD4+ T cells, suggesting a protective role (59). MELK thus supports tumor growth and an immune-evasive environment, complicating immunotherapy. Moreover, inhibitors such as OTSSP167 (phase I) and MELK-T1 show activity, but toxicity and off-target effects remain concerns, and therefore, safer options are needed (15, 60).

The study explored synthetic and natural MELK inhibitors. Docking indicated that OTSSP167 binds strongly to the MELK active site. G2/M inhibitors such as Onvansertib, Adavosertib, and Volasertib also engage MELK, suggesting an interaction between MELK signaling and mitotic checkpoints. Natural flavonoids, especially hesperidin and eriocitrin, bound MELK more strongly than OTSSP167; other flavonoids (narirutin, naringin, neohesperidin, rutin) also showed higher affinity. Molecular dynamics support stable binding of hesperidin. Combining flavonoids with MELK inhibitors may enhance efficacy and reduce toxicity, offering a potential strategy for LUAD therapy. ceRNA networks represent an additional layer of post-transcriptional regulation in which lncRNAs and mRNAs compete for shared miRNAs, thereby influencing gene expression (18). Within this framework, MELK may be regulated by lncRNA-miRNA-mRNA interactions, highlighting the potential role of ceRNA crosstalk in its dysregulation in LUAD. Moreover, in several cancer types, MELK has been shown to participate in ceRNA networks that influence tumor proliferation and metastasis. However, the present study extends this concept to LUAD by integrating transcriptional and post-transcriptional regulatory layers and identifying the TMPO-AS1/hsa-let-7b-5p/MELK–FOXM1 regulatory axis. In LUAD, hsa-let-7b-5p is downregulated and linked to poor prognosis; MELK and FOXM1 are predicted targets of this miRNA, suggesting that reduced let-7b-5p activity drives their co-upregulation. TMPO-AS1 likely acts as a sponge for let-7b-5p, supporting MELK-FOXM1 signaling. This TMPO-AS1/hsa-let-7b-5p/MELK-FOXM1 regulatory loop provides a mechanistic explanation for coordinated transcriptional and post-transcriptional dysregulation in LUAD progression and immune resistance. Collectively, the novelty of this study lies in the integrated identification of the MELK–FOXM1 regulatory relationship and the TMPO-AS1/hsa-let-7b-5p/MELK–FOXM1 ceRNA axis in LUAD, together with their association with the tumor immune microenvironment and prognostic features, followed by experimental validation of the proposed regulatory components. These findings provide a foundation for further establishing the functional significance of the TMPO-AS1/hsa-let-7b-5p/MELK–FOXM1 regulatory axes and exploring their therapeutic potential in LUAD.

## 5 Conclusion

Our study highlights MELK as a crucial oncogenic and immunomodulatory driver in lung adenocarcinoma (LUAD) and shows that it is regulated by the FOXM1-TMPO-AS1/hsa-let-7b-5p ceRNA axis. Dysregulation of this network promotes cell-cycle progression and epithelial-mesenchymal transition (EMT), fostering an immunosuppressive microenvironment. MELK serves as both a prognostic biomarker and a therapeutic target. Proposed intervention strategies include enhancing hsa-let-7b-5p activity to inhibit MELK and FOXM1, targeting TMPO-AS1 with siRNA and antisense oligonucleotides, and combining natural MELK inhibitors such as hesperidin with selective inhibitors like OTSSP167 to improve kinase blockade. These strategies reveal the therapeutic potential of the ceRNA axis and provide a basis for future studies in LUAD.

## Data Availability

The original contributions presented in the study are included in the article/**Supplementary Material**. Further inquiries can be directed to the corresponding author.

## Glossary

MELK: Maternal Embryonic Leucine Zipper Kinase
NSCLC: Non-Small Cell Lung Cancer
LUSC: Lung Squamous Cell Carcinoma
LUAD: Lung Adenocarcinoma
GO: Gene Ontology
TIMER 2.0: Tumor Immune Estimation Resource 2.0
GSCA: Gene Set Cancer Analysis
UALCAN: University of Alabama at Birmingham Cancer Data Analysis Portal
TCGA: The Cancer Genome Atlas
KMP/KM Plotter: Kaplan-Meier Plotte
OS: Overall Survival
CI: Confidence Interval
HR: Hazard Ratio
DGE: Differential Gene Expression
LCE: Lung Cancer Explorer
CancerSEA: Cancer Single Cell Functional States Atlas
ENCORI: Encyclopedia of RNA Interactomes
miRTarBase/miRTARGET: miRNA–Target Interaction Database
HPA: Human Protein Atlas
OncoDB: Integrated Cancer Genomics Database
GEPIA2: Gene Expression Profiling Interactive Analysis v2
TCGAnalyzeR: TCGA Visualization and Analysis Tool
TNMplot: Tumor-Node-Metastasis Plot Database
PPI: Protein-Protein Interaction
STRING: Search Tool for the Retrieval of Interacting Genes/Proteins
SIGNOR: Signaling Network Open Resource
FOXM1: Forkhead Box M1
ROC: Receiver Operating Characteristic
AUC: Area Under the Curve
UMAP: Uniform Manifold Approximation and Projection
DSV: Differential Signature Visualization
TF: Transcription Factor
miRNA: MicroRNA
RNA: Ribonucleic Acid
MiRWalk: miRNA Target Prediction Database
RNA22v2: RNA22 Version 2 miRNA Target Prediction Tool
CancerMIRNome: Pan-Cancer miRNA Expression Database
Enricher: Gene Set Enrichment Analysis Tool
MKI67: Marker of Proliferation Ki-67
EMT: Epithelial-Mesenchymal Transition
NK: Natural Killer Cells
CD8+: Cluster of Differentiation 8 (Cytotoxic T Cells)
CD4+: Cluster of Differentiation 4 (Helper T Cells)
HSC: Hematopoietic Stem Cell
MDSC: Myeloid-Derived Suppressor Cells
CAF: Cancer-Associated Fibroblasts.
