# Supplementary material for: Prognostic significance of the MELK/TMPO-AS1/hsa-let-7b-5p network in lung adenocarcinoma
Source: Front Oncol. 2026 Aug 31;16:1956981. doi: 10.3389/fonc.2026.1956981 (PMC13572222; doi:10.3389/fonc.2026.1956981)
Supplement: Supplementary file 1 [file Supplementaryfile1.docx]

**Title:** Prognostic Significance of the MELK/TMPO-AS1/hsa-let-7b-5p Network in Lung Adenocarcinoma

**Authors:**

Kritika Jangir^1^, kritika.2501049204@muj.manipal.edu

Niranjan Kumar^1^, niranjansinkuswaha@gmail.com

Chainsee Saini^1^, chainsee.2410160002@muj.manipal.edu

Prerna Vats^1^, prerna.23fs30sbs00028@muj.manipal.edu

Bhavika Baweja^1^, bhavika.2410160001@muj.manipal.edu

Prachi Patidar^1^, prachi.2501049206@muj.manipal.edu

Rashi Soni^1^, rashi.soni@jaipur.manipal.edu

Husen Lakdawala^2^, husen.lakdawala122010@marwadiuniversity.ac.in

Jaikee Kumar Singh^2^, jaikee.singh@marwadieducation.edu.in

Rajeev Nema^1^*, rajeev.nema@jaipur.manipal.edu

**Authors’ Affiliation:**

^1^Department of Biosciences, Manipal University Jaipur, Dehmi Kalan, Jaipur-Ajmer Expressway, Jaipur, Rajasthan, 303007, India

^2^Department of Bioinformatics, Marwadi University, Rajkot, Gujarat, India, 360003

**Address for Correspondence:**

*Dr. Rajeev Nema, PhD

Associate Professor, Department of Biosciences Manipal University Jaipur, Dehmi Kalan, Jaipur-Ajmer Expressway, Jaipur, Rajasthan, 303007, India

E-mail: [rajeev.nema@jaipur.manipal.edu](mailto:rajeev.nema@jaipur.manipal.edu)

**Supplementary Figure Legends**

**Supplementary Figure 1. Chromosomal localization and pan-cancer expression analysis of MELK. (A)** Chromosomal localization of the MELK gene was identified using the GeneLoc. Comparative MELK expression across TCGA cancers using multiple databases: **(B)** TIMER, **(C)** GSCA, and **(D)** UALCAN.

**Supplementary Figure 2. MELK expression in LUAD across multiple datasets.** Expression profiling of MELK in LUAD tumors vs. normal **(A)** UALCAN (tumor = 515, normal = 59), **(B)** ENCORI (tumor = 526, normal = 59), **(C)** ONCODB (LUAD = 540, normal = 59), **(D)** GEPIA (tumor = 483, normal = 347), and **(E)** LCE (tumor = 517, normal = 59). **(F)** UALCAN analysis shows MELK expression in LUAD based on nodal metastasis status.

**Supplementary Figure 3. Correlation and expression analysis of FOXM1 and MELK in LUAD. (A-F)** CancerSEA showing correlations between MELK expression and cell cycle, proliferation, DNA repair, DNA damage, invasion, and EMT activities. Expression profiling of FOXM1 in LUAD tumor vs. normal using **(G)** UALCAN and **(H)** ENCORI. **(I)** FOXM1 overexpression drives MELK transcriptional upregulation in LUAD tissue. **(J)** STRING database-PPI network of MELK-associated genes. **(K)** FOXM1 expression in LUAD based on nodal metastasis status using UALCAN.

**Supplementary Figure 4. Immune response prediction and gene expression and immune cell infiltration in TCGA-LUAD.** ROC curves showing predictive performance of Gene for immune response in **(A)** MELK and **(B)** FOXM1. Spearman correlation heatmap of gene expression and immune cell infiltration in TCGA-LUAD from the **(C)** GSCA database.

**Supplementary Figure 5. Single-cell analysis of MELK and FOXM1 in LUAD.** (**A-B)** UMAP visualization of cell clusters and annotated cell types. **(C)** Mean expression of cell types. Cell trajectory analysis showing developmental relationships among **(D)** clusters and **(E)** cell types using CellTracer.

**Supplementary Figure 6. Molecular interaction analysis of MELK with chemotherapeutic agents and mitotic inhibitors.** 2D interaction diagrams of MELK with **(A)** OTSSP167, **(B)** Paclitaxel, **(C)** Docetaxel, **(D)** Vinblastine, **(E)** Vincristine, **(F)** Nocodazole, **(G)** Barasertib, **(H)** Volasertib, and **(I)** Onvansertib.

**Supplementary Figure 7. Molecular interaction analysis of MELK with mitotic inhibitors and natural compounds.** 2D interaction diagrams of MELK with **(A)** Alisertib, **(B)** RP_6306, **(C)** Adavosertib, **(D)** Prexasertib, **(E)** UCN02, **(F)** AZD7762, **(G)** Hesperidin, and **(H)** Naringin.

**Supplementary Figure 8. Molecular interaction analysis of MELK with natural compounds.** 2D interaction diagrams of MELK with **(A)** Neohesperidin, **(B)** Hesperetin, **(C)** Narirutin, **(D)** Eriocitrin, **(E)** Quercetin, **(F)** Kaempferol, **(G)** Fisetin, **(H)** Isorhamnetin, **(J)** Rutin, and **(K)** Myricetin.

**Supplementary Figure 9. Molecular interaction analysis of MELK with chemotherapeutic agents and mitotic inhibitors.** 3D interaction diagrams of MELK with **(A)** Paclitaxel, **(B)** Docetaxel, **(C)** Vinblastine, **(D)** Vincristine, **(E)** Nocodazole, **(F)** Barasertib, **(G)** Volasertib, and **(H)** Onvansertib**.**

**Supplementary Figure 10. Molecular interaction analysis of MELK with mitotic inhibitors and natural compounds.** 3D interaction diagrams of MELK with **(A)** Alisertib, **(B)** RP_6306, **(C)** Adavosertib, **(D)** Prexasertib, **(E)** UCN02, **(F)** AZD7762, **(G)** Naringin.

**Supplementary Figure 11. Molecular interaction analysis of MELK with a natural compound.** 3D interaction diagrams of MELK with **(A)** Neohesperidin, **(B)** Hesperetin, **(C)** Narirutin, **(D)** Eriocitrin, **(E)** Quercetin, **(F)** Kaempferol, **(G)** Fisetin, **(H)** Isorhamnetin, **(J)** Rutin, and **(K)** Myricetin.

**Supplementary Figure 12. Integrative Analysis of miRNA Gene Interactions, Expression Correlations, and Clinical Significance in LUAD. (A)** FOXM1 activation and reduced hsa-let-7b-5p lead to increased MELK levels in LUAD. **(B)** miRNet showing miRNA-gene interaction network. ENCORI showing correlations between miRNA and target gene expression in LUAD, **(C)** hsa-let-7b-5p vs. MELK, **(D)** hsa-let-7b-5p vs. FOXM1, **(E)** hsa-mir-29c-3p vs. MELK, and **(F)** hsa-mir-29c-3p vs. FOXM1. Survival analysis curves of miRNAs using KM Plotter **(G)** hsa-let-7b and **(H)** hsa-mir-29c. **(I-L)** ROC curves for miRNAs in LUAD. **(M)** Expression of hsa-let-7b in LUAD based on nodal metastasis status by UALCAN.

**Supplementary Figure 13. Expression and Interaction Analysis of TMPO-AS1 in LUAD.** (**A)** Comparative TMPO-AS1 expression across cancer using lncRNAfunc 2.0 database. Expression profiling of TMPO-AS1 in LUAD and normal tissues analyzed using **(B)** OncoDB and **(C)** UALCAN. **(D)** Comparative analysis of TMPO-AS1 expression between female smokers and non-smokers in LUAD by using LCE. **(E)** miRNA-mRNA-lncRNA interaction network of TMPO-AS1, FOXM1, and MELK constructed using miRNet. **(F)** Cytoscape-based regulatory network.

**Supplementary Table 1: MELK involvement in cellular process**

| **GO ID** | **Qualified GO term** | **Evidence** |
| --- | --- | --- |
| [GO:0000086](https://amigo.geneontology.org/amigo/term/GO:0000086) | Involved in G2/M Transition of Mitotic Cell Cycle | TAS |
| [GO:0006338](https://amigo.geneontology.org/amigo/term/GO:0006338) | Chromatin Remodeling | IEA |
| [GO:0006468](https://amigo.geneontology.org/amigo/term/GO:0006468) | Protein Phosphorylation | IEA |
| [GO:0006915](https://amigo.geneontology.org/amigo/term/GO:0006915) | Involved in Apoptotic Process | IDA, IEA |
| [GO:0008283](https://amigo.geneontology.org/amigo/term/GO:0008283) | Involved in Cell Population Proliferation | IDA, IEA |
| [GO:0008631](https://amigo.geneontology.org/amigo/term/GO:0008631) | Acts Upstream of or Within Intrinsic Apoptotic Signaling Pathway in Response to Oxidative Stress | IEA |
| [GO:0030097](https://amigo.geneontology.org/amigo/term/GO:0030097) | Involved in Hemopoiesis | ISS |
| [GO:0043065](https://amigo.geneontology.org/amigo/term/GO:0043065) | Involved in Positive Regulation of Apoptotic Process | ISS, IEA |
| [GO:0046777](https://amigo.geneontology.org/amigo/term/GO:0046777) | Involved in Protein Autophosphorylation | IDA |
| [GO:0061351](https://amigo.geneontology.org/amigo/term/GO:0061351) | Involved in Neural Precursor Cell Proliferation | ISS, IEA |

**Supplementary Table 2: Prognostic Significance of MELK in Lung Cancer**

| **S.NO.** | **Gene** | **Index** | | **Patient Number** | **Hazard Ratio** | **CI** | **Log(P)** | **Low Expression Cohort (Months)** | **High Expression Cohort (Months)** |
| --- | --- | --- | --- | --- | --- | --- | --- | --- | --- |
| 1 | MELK | OS | | 2166 | 1.63 | 1.44-1.84 | 1.10e-15 | 95 | 47.63 |
|  |  | FP | | 1252 | 1.82 | 1.53-2.16 | 3.90e-12 | 31.41 | 11 |
|  |  | PPS | | 477 | 1.2 | 0.98-1.48 | 0.085 | 17.67 | 10.78 |
| OS + Histology | | | | | | | | | |
| 2 |  | Adenocarcinoma | | 1161 | 1.58 | 1.33-1.88 | 1.70e-07 | 105 | 61.3 |
|  | MELK | Squamous cell Carcinoma | | 780 | 1.03 | 0.85-1.25 | 0.74 | 57 | 54 |
| OS + LUAD + Stages | | | | | | | | | |
| 3 | MELK | Stage1 | | 370 | 2.07 | 1.38-3.12 | 0.00035 | 76 | 40.3 |
|  |  | Stage2 | | 136 | 0.76 | 0.47-1.23 | 0.26 | 49 | 68.67 |
|  |  | Stage3 | | 24 | 2.1 | 0.73-6.05 | 0.16 | 52 | 26.09 |
| OS + LUAD + Gender | | | | | | | | | |
| 4 | MELK |  | | 566 | 1.47 | 1.16-1.86 | 0.0012 | 89 | 52 |
|  |  | Male | |  |  |  |  |  |  |
|  |  |  | | 537 | 1.73 | 1.31-2.29 | 8.10e-05 | 110.27 | 75.43 |
|  |  | Female | |  |  |  |  |  |  |
| OS + LUAD + Smoking History | | | | | | | | | |
| 5 | MELK | Smoker | | 546 | 1.54 | 1.91-2.01 | 0.001 | 91 | 65 |
|  |  | Non-Smoker | | 192 | 1.59 | 0.87-2.88 | 0.13 | 76 | 56.5 |
| OS + LUAD + Smoking History + Gender | | | | | | | | | |
| 6 | MELK | Smoker | Male | 319 | 1.29 | 0.91-1.82 | 0.15 | 79 | 81 |
|  |  |  | Female | 227 | 1.5 | 1.01-2.23 | 0.042 | 95 | 65 |

| **S.NO.** | **G2/M transition phase regulators** | **MELK**  **(R-value)** |
| --- | --- | --- |
| 1 | CDK1 | 0.865 |
| 2 | CCNB1 | 0.840 |
| 3 | PLK1 | 0.836 |
| 4 | CDC25C | 0.843 |

**Supplementary Table 3: Correlation of MELK with key G2/M transition phase regulator**

| **S.NO.** | **GENE** | **EMT MARKER** | **R VALUE** | **P- VALUE** |
| --- | --- | --- | --- | --- |
| 1. | MELK | TWIST1 | 0.358 | 2.47e-17 |
| 2. | MELK | SNAI1 | 0.334 | 3.53e-15 |
| 3. | MELK | SNAI2 | 0.199 | 4.32-06 |
| 4. | MELK | VIM | -0.091 | 3.62e-02 |
| 5. | MELK | CDH2 | 0.271 | 2.68e-10 |
| 6. | MELK | SOX10 | 0.000 | 1.00e+00 |
| 7. | MELK | CDH1 | -0.063 | 1.51E-01 |
| 8. | MELK | DSP | 0.347 | 2.60E-16 |
| 9. | MELK | TJP1 | -0.101 | 2.06E-02 |
| 10 | MELK | FOXC2 | -0.028 | 5.24E-01 |
| 11. | MELK | GSC | 0.076 | 8.00E-02 |
| 12. | MELK | FN1 | 0.225 | 1.86E-07 |
| 13. | MELK | MMP2 | -0.029 | 5.09E-01 |
| 14. | MELK | MMP3 | 0.188 | 1.45E-05 |
| 15. | MELK | MMP9 | 0.238 | 3.4E-08 |

**Supplementary Table 4: Correlation of EMT Markers with MELK**

**Supplementary Table 5: Prognostic Significance of MELK Associated EMT Markers**

| **S.NO.** | **Gene** | **Index** | **Patient Number** | **Hazard Ratio** | **CI** | **Log(P)** | **Low Expression Cohort (Months)** | **High Expression Cohort (Months)** |
| --- | --- | --- | --- | --- | --- | --- | --- | --- |
| **1.** | TWIST1 | OS | 2166 | 1.24 | 1.1-1.39 | 0.00045 | 80 | 59.53 |
| **2.** | SNAI1 | OS | 2166 | 1.27 | 1.13-1.43 | 6.8e-05 | 78.9 | 57 |
| **Histology** | | | | | | | | |
| **1.** | TWIST1 | OS + LUAD | 1161 | 1.21 | 1.02-1.44 | 0.029 | 96 | 74 |
| **2.** | SNAI1 | OS + LUAD | 1161 | 1.37 | 1.16-1.63 | 0.00027 | 99 | 69.93 |

**Supplementary Table 6: Heterogeneous Model of MELK in LUAD**

| **S.NO.** | **GENE** | **R-VALUE**  **(UALCAN)** | **R-VALUE**  **(ENCORI)** |
| --- | --- | --- | --- |
| **Positive Correlators** | | | |
| 1. | CKAP2L | 0.83 | 0.911 |
| 2. | KIF4A | 0.82 | 0.902 |
| 3. | NCAPG | 0.82 | 0.903 |
| 4. | CCNA2 | 0.82 | 0.888 |
| 5. | CDCA5 | 0.82 | 0.882 |
| 6. | TTK | 0.81 | 0.903 |
| 7. | BUB1 | 0.81 | 0.901 |
| 8. | SKA1 | 0.81 | 0.891 |
| 9. | CCNB1 | 0.81 | 0.840 |
| 10. | DEPDC1 | 0.81 | 0.884 |
| **Negative Correlators** | | | |
| 1. | NAPSA | -0.45 | -0.527 |
| 2. | TMEM125 | -0.42 | -0.512 |
| 3. | C17orf108 (LYRM9) | -0.42 | -0.517 |
| 4. | C16orf89 | -0.42 | -0.636 |
| 5. | SELENBP1 | -0.42 | -0.619 |
| 6. | RILP | -0.42 | -0.467 |
| 7. | LMF1 | -0.41 | -0.511 |
| 8. | SLC22A3 | -0.41 | -0.542 |
| 9. | SFTPB | -0.41 | -0.518 |
| 10. | CD81 | -0.41 | -0.520 |

**Table 7: Correlation of MELK with Corelated genes by SIGNOR**

| **S.NO** | **GENE** | **GENE**  **(Signor)** | **R-Value**  **SIGNOR** | **R-value ENCHORI** | **R-VLAUE ONCODB** | **GEPIA**  **(R-value)** |
| --- | --- | --- | --- | --- | --- | --- |
| 1. | MELK | FOXM1 | 0.504 | 0.834 | 0.836 | 0.854 |
| 2. |  | SQSTM1 | 0.2 | -0.130 | -0.0874 | -0.0752 |
| 3. |  | PTK2 | 0.2 | 0.115 | 0.1564 | 0.18 |
| 4. |  | EZH2 | 0.33 | 0.746 | 0.7516 | 0.769 |
| 5. |  | FOXO3 | 0.363 | -0.097 | -0.008 | 0.0363 |
| 6. |  | FOXO1 | 0.257 | -0.256 | -0.187 | -0.232 |
| 7. |  | PDPK1 | 0.267 | -0.263 | -0.129 | -0.0638 |
| 8. |  | STK11 | 0.2 | -0.029 | 0.026 | 0.0489 |
| 9. |  | CDC25B | 0.535 | 0.287 | 0.325 | 0.345 |

**Supplementary Table 8: Survival Analysis of FOXM1**

| **S.NO.** | **Gene** | **Index** | | **Patient Number** | **Hazard Ratio** | **CI** | **Log(P)** | **Low Expression Cohort (Months)** | **High Expression Cohort (Months)** |
| --- | --- | --- | --- | --- | --- | --- | --- | --- | --- |
| 1 | FOXM1 | OS | | 2166 | 1.85 | 1.64-2.09 | <1E-16 | 96 | 44 |
|  |  | FP | | 1252 | 1.87 | 1.58-2.23 | 3.6e-13 | 32 | 11 |
|  |  | PPS | | 477 | 1.42 | 1.15-1.75 | 0.00095 | 20 | 9 |
| OS + Histology | | | | | | | | | |
| 2 | FOXM1 | Adenocarcinoma | | 1161 | 2.11 | 1.76-2.51 | <1e-16 | 108.97 | 48.9 |
|  |  | Squamous cell Carcinoma | | 780 | 1.26 | 1.04-1.52 | 0.021 | 63.03 | 43.83 |
| OS + LUAD + Stages | | | | | | | | | |
| 3 | FOXM1 | Stage1 | | 370 | 3.13 | 2.03-4.83 | 5.7e-08 | 110.27 | 38.1 |
|  |  | Stage2 | | 136 | 1.59 | 0.98-2.58 | 0.056 | 88.7 | 34.33 |
|  |  | Stage3 | | 24 | 1 | 0.36-2.76 | 0.99 | 33.83 | 37.97 |
| OS + LUAD + Gender | | | | | | | | | |
| 4 | FOXM1 | Male | | 566 | 1.83 | 1.44-2.32 | 4.8e-07 | 105 | 45 |
|  |  | Female | | 537 | 2.35 | 1.77-3.13 | 1.3e-09 | 117.33 | 65 |
| OS + LUAD + Smoking History | | | | | | | | | |
| 5 | FOXM1 | Smoker | | 546 | 2.2 | 1.68-2.88 | 5.3e-09 | 116 | 52 |
|  |  | Non-Smoker | | 192 | 2.21 | 1.19-4.12 | 0.01 | 76 | 49.4 |
| OS + LUAD + Smoking History + Gender | | | | | | | | | |
| 6 | FOXM1 | Smoker | Male | 319 | 2.34 | 1.63-3.36 | 2.2e-06 | 61 | 21.3 |
|  |  |  | Female | 227 | 2.08 | 1.38-3.14 | 0.00035 | 116 | 52 |
|  |  | Non-smokers | Male | 31 | 4.5 | 0.93-21.77 | 0.04 | - | - |
|  |  |  | Female | 161 | 2.39 | 1.17-4.87 | 0.014 | 76 | 52 |

**Supplementary Table 9: Heterogenous Model Associated with TIME**

| S.NO. | GENE | CD8+T CELL (Rho value) | CD4+T CELL (Rho value) | B CELLS | Neutrophil | Monocyte | Macrophage  (M2) | DC |
| --- | --- | --- | --- | --- | --- | --- | --- | --- |
| 1. | MELK | -0.231 | -0.334 | -0.337 | -0.283 | -0.203 | -0.39 | -0.327 |
| 2. | CKAP2L | -0.241 | -0.289 | -0.35 | -0.261 | -0.16 | -0.411 | -0.265 |
| 3. | KIF4A | -0.295 | -0.303 | -0.339 | -0.253 | -0.197 | -0.408 | -0.345 |
| 4. | NCAPG | -0.246 | -0.324 | -0.331 | -0.312 | -0.22 | -0.391 | -0.32 |
| 5. | CCNA2 | -0.239 | -0.3 | -0.355 | -0.325 | -0.164 | -0.404 | -0.265 |
| 6. | CDCA5 | -0.266 | -0.346 | -0.312 | -0.277 | -0.199 | -0.367 | -0.367 |
| 7. | TTK | -0.208 | -0.333 | -0.365 | -0.325 | -0.216 | -0.376 | -0.333 |
| 8. | BUB1 | -0.254 | -0.301 | -0.331 | -0.289 | -0.197 | -0.397 | -0.299 |
| 9. | SKA1 | -0.214 | -0.342 | -0.33 | -0.32 | -0.235 | -0.333 | -0.383 |
| 10. | CCNB1 | -0.266 | -0.34 | -0.355 | -0.317 | -0.204 | -0.319 | -0.315 |
| 11. | DEPDC1 | -0.226 | -0.34 | -0.362 | -0.301 | -0.174 | -0.353 | -0.299 |
| 12. | NAPSA | 0.229 | 0.347 | 0.226 | 0.443 | 0.274 | 0.367 | 0.392 |
| 13. | TMEM125 | 0.312 | 0.213 | 0.224 | 0.366 | 0.183 | 0.434 | 0.14 |
| 14. | C17orf108 (LYRM9) | 0.255 | 0.174 | 0.236 | 0.222 | 0.11 | 0.308 | 0.202 |
| 15. | C16orf89 | 0.28 | 0.257 | 0.246 | 0.44 | 0.308 | 0.417 | 0.347 |
| 16. | SELENBP1 | 0.281 | 0.201 | 0.26 | 0.411 | 0.229 | 0.35 | 0.211 |
| 17. | RILP | 0.242 | 0.278 | 0.222 | 0.15 | 0.308 | 0.25 | 0.309 |
| 18. | LMF1 | 0.023 | 0.24 | 0.425 | 0.342 | 0.141 | 0.126 | 0.158 |
| 19. | SLC22A3 | 0.165 | 0.171 | 0.211 | 0.393 | 0.229 | 0.243 | 0.237 |
| 20 | SFTPB | 0.235 | 0.205 | 0.212 | 0.425 | 0.245 | 0.336 | 0.226 |
| 21. | CD81 | 0.132 | 0.201 | 0.2 | 0.225 | 0.192 | 0.155 | 0.297 |

| S.NO. | GENE | NK | Mast cell | CAF | CLP | CMP | Endothelial | eosinophil | Granulocyte monocyte progenitor |
| --- | --- | --- | --- | --- | --- | --- | --- | --- | --- |
| 1. | MELK | -0.147 | -0.468 | 0.232 | 0.319 | -0.23 | -0.316 | -0.15 | -0.181 |
| **Positive Correlators** | | | | | | | | | |
| 2. | CKAP2L | -0.195 | -0.46 | 0.28 | 0.365 | -0.23 | -0.231 | -0.13 | -0.099 |
| 3. | KIF4A | -0.143 | -0.467 | 0.288 | 0.296 | -0.233 | -0.265 | -0.123 | -0.207 |
| 4. | NCAPG | -0.214 | -0.492 | 0.209 | 0.392 | -0.238 | -0.34 | -0.174 | -0.218 |
| 5. | CCNA2 | -0.263 | -0.469 | 0.219 | 0.398 | -0.221 | -0.346 | -0.14 | -0.185 |
| 6. | CDCA5 | -0.129 | -0.481 | 0.277 | 0.296 | -0.208 | -0.28 | -0.137 | -0.221 |
| 7. | TTK | -0.183 | -0.463 | 0.209 | 0.361 | -0.215 | -0.32 | -0.138 | -0.184 |
| 8. | BUB1 | -0.19 | -0.479 | 0.227 | 0.36 | -0.227 | -0.286 | -0.141 | -0.156 |
| 9. | SKA1 | -0.212 | -0.493 | 0.193 | 0.339 | -0.256 | -0.291 | -0.124 | -0.24 |
| 10. | CCNB1 | -0.279 | -0.48 | 0.173 | 0.447 | -0.233 | -0.381 | -0.161 | -0.239 |
| 11. | DEPDC1 | -0.235 | -0.472 | 0.224 | 0.457 | -0.213 | -0.317 | -0.145 | -0.171 |
| **Negative Correlators** | | | | | | | | | |
| 12. | NAPSA | 0.126 | 0.467 | -0.177 | -0.198 | 0.197 | 0.313 | 0.049 | 0.349 |
| 13. | TMEM125 | 0.223 | 0.417 | -0.315 | -0.278 | 0.167 | 0.25 | 0.072 | 0.165 |
| 14. | C17orf108 (LYRM9) | 0.269 | 0.377 | -0.178 | -0.214 | 0.098 | 0.225 | 0.073 | 0.08 |
| 15. | C16orf89 | 0.244 | 0.517 | -0.241 | -0.303 | 0.213 | 0.343 | 0.065 | 0.298 |
| 16. | SELENBP1 | 0.28 | 0.455 | -0.289 | -0.365 | 0.206 | 0.387 | 0.11 | 0.2 |
| 17. | RILP | 0.042 | 0.324 | -0.255 | -0.23 | 0.154 | 0.14 | 0.094 | 0.112 |
| 18. | LMF1 | 0.441 | 0.32 | -0.034 | -0.507 | 0.166 | 0.27 | 0.168 | 0.082 |
| 19. | SLC22A3 | 0.222 | 0.413 | -0.185 | -0.288 | 0.214 | 0.375 | 0.055 | 0.261 |
| 20 | SFTPB | 0.04 | 0.35 | -0.233 | -0.266 | 0.109 | 0.389 | 0.129 | 0.22 |
| 21. | CD81 | 0.25 | 0.294 | -0.004 | -0.441 | 0.178 | 0.411 | 0.118 | 0.129 |

| S.NO. | GENE | HSC | T-CELL FOLICULAR HELPER | T CELL GAMMA DELTA | T CELL NK | MYLOIED DERIVED SUPPRESOR CELL |
| --- | --- | --- | --- | --- | --- | --- |
| 1. | MELK | -0.668 | 0.127 | 0.086 | -0.293 | 0.646 |
| **Positive Correlators** | | | | | | |
| 2. | CKAP2L | -0.636 | 0.079 | 0.043 | -0.306 | 0.61 |
| 3. | KIF4A | -0.659 | 0.113 | 0.032 | -0.307 | 0.669 |
| 4. | NCAPG | -0.704 | 0.117 | 0.072 | -0.316 | 0.682 |
| 5. | CCNA2 | -0.678 | 0.101 | 0.084 | -0.313 | 0.643 |
| 6. | CDCA5 | -0.684 | 0.119 | 0.04 | -0.304 | 0.697 |
| 7. | TTK | -0.658 | 0.114 | 0.086 | -0.327 | 0.65 |
| 8. | BUB1 | -0.683 | 0.12 | 0.067 | -0.308 | 0.637 |
| 9. | SKA1 | -0.661 | 0.139 | 0.086 | -0.317 | 0.686 |
| 10. | CCNB1 | -0.687 | 0.042 | 0.071 | -0.291 | 0.719 |
| 11. | DEPDC1 | -0.64 | 0.059 | 0.091 | -0.348 | 0.681 |
| **Negative Correlators** | | | | | | |
| 12. | NAPSA | 0.54 | -0.064 | -0.067 | 0.341 | -0.546 |
| 13. | TMEM125 | 0.492 | -0.01 | -0.064 | 0.177 | -0.479 |
| 14. | C17orf108 (LYRM9) | 0.478 | 0.079 | -0.002 | 0.207 | -0.455 |
| 15. | C16orf89 | 0.607 | -0.052 | -0.086 | 0.291 | -0.587 |
| 16. | SELENBP1 | 0.543 | 0.052 | -0.143 | 0.177 | -0.575 |
| 17. | RILP | 0.415 | -0.019 | -0.084 | 0.335 | -0.492 |
| 18. | LMF1 | 0.367 | 0.1 | -0.161 | 0.299 | -0.401 |
| 19. | SLC22A3 | 0.521 | -0.026 | -0.147 | 0.119 | -0.496 |
| 20 | SFTPB | 0.541 | 0.005 | -0.132 | 0.167 | -0.512 |
| 21. | CD81 | 0.523 | -0.026 | -0.129 | 0.218 | -0.487 |

**Supplementary Table 10: Binding Affinity of MELK with Selective Inhibitor, Natural compounds, and Chemotherapeutic drugs**

| **MOLECULE** |  | **INHIBITOR** | **BINDING AFFINITY(Kcal/mol)** |
| --- | --- | --- | --- |
| MELK | Direct Inhibitor | OTSSP167 | -9.672 |
|  | G2/M specific Chemotherapeutic Drugs | Docetaxel | -7.549 |
|  |  | Paclitaxel | -7.753 |
|  |  | Vinblastin | -7.642 |
|  |  | Vincristine | -6.93 |
|  |  | Nocodazole | -7.247 |
|  |  | Volasertib | -9.264 |
|  |  | Onvansertib | -9.374 |
|  |  | Alisertib | -8.993 |
|  |  | Barasertib | -8.906 |
|  |  | RP-6306 | -8.07 |
|  |  | Adavosertib | -9.296 |
|  |  | Prexasertib | -7.26 |
|  |  | UCN02 | -8.057 |
|  |  | AZD7762 | -8.436 |
|  | Natural Compounds | Hesperedin | -10.05 |
|  |  | Quercetin | -8.411 |
|  |  | Naringin | -9.556 |
|  |  | Neohesperidin | -9.556 |
|  |  | Hespertin | -8.37 |
|  |  | Narirutin | -9.866 |
|  |  | Eriocitrin | -10.03 |
|  |  | Kaempferol | -8.367 |
|  |  | Myricetin | -8.214 |
|  |  | Rutin | -9.361 |
|  |  | Isorhamnetin | -8.339 |
|  |  | Fisetin | -8.545 |

**Supplementary Table 11: Comparative MD analysis of MELK complexes**

| **Parameter** | **MELK-OTSSP167** | **MELK-Hesperidin** |
| --- | --- | --- |
| Backbone RMSD (nm) | 0.18 | 0.28 |
| Backbone RMSF (nm) | 0.16 | 0.16 |
| Radius of gyration (Rg, nm) | 2.16 | 2.16 |
| Solvent-accessible surface area (SASA, nm²) | 169 | 164 |
| Hydrogen bonds | 3 | 5 |

**Supplementary Table 12: MELK/FOXM1 associated miRNAs**

| **S.No.** | **miRNA** | **Expression Level** | **P-Value** |
| --- | --- | --- | --- |
| 1 | hsa-let-7b-5p | Downregulated | 5.75e-03 |
| 2 | hsa-miR-15a-5p | Up regulated | 4.83e-09 |
| 3 | hsa-miR-16-5p | Up regulated | 1.10e-03 |
| 4 | hsa-miR-17-5p | Up regulated | 1.62e-12 |
| 5 | hsa-miR-20a-5p | Up regulated | 1.62e-12 |
| 6 | hsa-miR-24-3p | Up regulated | 3.13e-03 |
| 7 | hsa-miR-26a-5p | Up regulated | 3.26e-12 |
| 8 | hsa-miR-98-5p | Down regulated | 8.51e-01 |
| 9 | hsa-miR-29b-3p | Up regulated | <1e-12 |
| 10 | hsa-miR-196a-5p | Up regulated | 1.57e-11 |
| 11 | hsa-miR-7-5p | Up regulated | 1.62e-12 |
| 12 | hsa-miR-34a-5p | Up regulated | 1.62e-12 |
| 13 | hsa-miR-181a-5p | Up regulated | 3.96e-02 |
| 14 | hsa-miR-203a-3p | Up regulated | 8.88e-16 |
| 15 | hsa-miR-200b-3p | Up regulated | 1.51e-09 |
| 16 | hsa-let-7g-5p | Downregulated | 1.59e-05 |
| 17 | hsa-miR-1-3p | NA | NA |
| 18 | hsa-miR-142-3p | Upregulated | <1e-12 |
| 19 | hsa-miR-29c-3p | Down regulated | 2.69e-01 |
| 20 | hsa-miR-375-3p | Upregulated | 1.33e-02 |
| 21 | hsa-miR-19a-3p | Upregulated | <1e-12 |
| 22 | hsa-miR-26b-5p | Upregulated | 7.17e-09 |
| 23 | hsa-miR-221-3p | Down regulated | 9.30e-01 |
| 24 | hsa-miR-138-5p | Down regulated | 2.04e-02 |
| 25 | hsa-miR-126-3p | Down regulated | 1.93e-01 |
| 26 | hsa-miR-193a-3p | Upregulated | 6.25e-02 |
| 27 | hsa-miR-155-5p | Upregulated | 3.80e-07 |
| 28 | hsa-miR-326 | Upregulated | 1.39e-01 |
| 29 | hsa-miR-429 | Upregulated | <1e-12 |
| 30 | hsa-miR-193b-3p | Upregulated | 1.50e-03 |
| 31 | hsa-miR-125a-3p | Upregulated | 5.33e-02 |

**Supplementary Table 13: miRNAs Correlation with MELK/FOXM1**

| **S.No.** | **miRNA** | **MELK(R-value)** | **FOXM1(R-value)** |
| --- | --- | --- | --- |
| 1. | hsa-let-7b-5p | -0.360 | -0.393 |
| 2. | hsa-miR-98-5p | 0.054 | -0.003 |
| 3. | hsa-let-7g-5p | -0.171 | -0.191 |
| 4. | hsa-miR-29c-3p | -0.393 | -0.406 |
| 5. | hsa-miR-221-3p | -0.049 | -0.075 |
| 6. | hsa-miR-138-5p | 0.070 | 0.020 |
| 7. | hsa-miR-126-3p | -0.078 | -0.123 |

**Supplementary Table 14: let-7 Series Survival Analysis**

| **S.NO.** | **Gene** | **Index** | **Patient Number** | **Hazard Ratio** | **CI** | **Log(P)** | **Low expression cohort (months)** | **High expression cohort (months)** |
| --- | --- | --- | --- | --- | --- | --- | --- | --- |
| 1. | hsa-let-7a | OS | 504 | 1.28 | 0.96-1.72 | 0.093 | 50.03 | 41.93 |
| 2. | hsa-let-7b | OS | 504 | 0.72 | 0.54-0.96 | 0.026 | 42.17 | 54.4 |
| 3. | hsa-let-7c | OS | 504 | 0.6 | 0.44-0.83 | 0.002 | 41.17 | 61 |
| 4. | hsa-let-7d | OS | 504 | 0.75 | 0.53-1.07 | 0.11 | 48.47 | 105.63 |
| 5. | hsa-let-7e | OS | 504 | 1.49 | 1.09-2.03 | 0.011 | 53.33 | 34.87 |
| 6. | hsa-let-7f | OS | 504 | 1.25 | 0.93-1.69 | 0.14 | 50.03 | 42.27 |
| 7. | hsa-let-7g | OS | 504 | 0.76 | 0.55-1.05 | 0.1 | 43.1 | 54.07 |
| 8. | hsa-let-7i | OS | 504 | 1.36 | 1-1.83 | 0.046 | 57.5 | 43.1 |
| 9. | hsa-miR-98 | OS | 504 | 0.82 | 0.61-1.11 | 0.19 | 42.17 | 50.93 |
| 10. | hsa-miR-202 | OS | 504 | 0.62 | 0.43-0.91 | 0.012 | 49.73 | 61 |

**Supplementary Table 15: Binding Affinities of hsa with MELK**

| **miRNA Name** | **Transcript Name** | **Target Site** | **Binding Energy** | **Folding Energy** | **Heteroduplex** |
| --- | --- | --- | --- | --- | --- |
| **hsa-let-7b-5p** | MELK | 1093 | -20.1 | -16.70 |  |
| **hsa-let-7c-5p** | MELK | 1093 | -15.1 | -12.70 |  |

**Supplementary Table 16: hsa-let-7b-5p associated lncRNAs**

| **Index** | **Name** | **P-value** | **Expression Profile** | **P-Value** |
| --- | --- | --- | --- | --- |
| 1. | MDC1-AS1 | 1.003e-15 | NA | 7.17e-09 |
| 2. | PLCG1-AS1 | 8.478e-15 | Downregulated | 9.24e-04 |
| 3. | GTF3C2-AS1 | 6.817e-14 | Upregulated | 2.4e-16 |
| 4. | SMARCA5-AS1 | 3.784e-12 | Downregulated | 6.80e-02 |
| 5. | PABPC4-AS1 | 1.699e-10 | Upregulated | 6.39e-07 |
| 6. | NFYC-AS1 | 1.048e-9 | Upregulated | 1.93e-21 |
| 7. | LINC00449 | 6.106e-9 | Upregulated | 7.47e-09 |
| 8. | EXOSC10-AS1 | 6.106e-9 | Upregulated | 1.18e-28 |
| 9. | LMLN-AS1 | 6.106e-9 | NA | 1.36e-08 |
| 10. | PITRM1-AS1 | 6.106e-9 | Upregulated | 4.41e-06 |
| 11. | MTOR-AS1 | 3.353e-8 | NA | 1.73e-04 |
| 12. | SMG7-AS1 | 3.353e-8 | Upregulated | 2.69e-13 |
| 13. | VPS9D1-AS1 | 3.353e-8 | Upregulated | 1.51e-61 |
| 14. | ODF2-AS1 | 1.732e-7 | Upregulated | 2.43e-06 |
| 15. | ERCC8-AS1 | 1.732e-7 | NA | 3.11e-07 |
| 16. | LINC02787 | 8.404e-7 | NA | NA |
| 17. | LINC01775 | 8.404e-7 | NA | 3.60e-36 |
| 18. | STARD7-AS1 | 8.404e-7 | Upregulated | 1.65e-10 |
| 19. | H2AZ1-DT | 8.404e-7 | Upregulated | 7.78e-08 |
| 20. S | RNF213-AS1 | 8.404e-7 | Downregulated | 2.79e-01 |
| 21. | TBL1XR1-AS1 | 0.000003819 | NA | 1.04e-04 |
| 22. | INO80-AS1 | 0.000003819 | Downregulated | 1.95e-02 |
| 23. | TMEM202-AS1 | 0.000003819 | Downregulated | 6.30e-01 |
| 24. | LINC02163 | 0.00001621 | NA | NA |
| 25. | SREBF2-AS1 | 0.00001621 | Upregulated | 1.81e-16 |
| 26. | LINC01357 | 0.00006412 | Upregulated | 5.62e-15 |
| 27. | LINC01234 | 0.00006412 | NA | 1.85e-12 |
| 28. | EMC1-AS1 | 0.00006412 | Upregulated | 1.90e-06 |
| 29. | XXYLT1-AS1 | 0.00006412 | NA | NA |
| 30. | SPIN4-AS1 | 0.00006412 | NA | 7.47e-02 |
| 31. | TAB3-AS2 | 0.00006412 | NA | NA |
| 32. | MYCBP2-AS1 | 0.00006412 | Downregulated | 1.53e-03 |
| 33. | PRMT5-AS1 | 0.00006412 | Upregulated | 1.59e-25 |
| 34. | LLPH-DT | 0.00006412 | Upregulated | 4.25e-04 |
| 35. | HLTF-AS1 | 0.00006412 | NA | NA |
| 36. | SMAD1-AS1 | 0.00006412 | NA | NA |
| 37. | LIX1-AS1 | 0.00006412 | Upregulated | 4.06e-04 |
| 38. | LINC01446 | 0.0002356 | NA | 1.25e-13 |
| 39. | IGBP1-AS2 | 0.0002356 | Upregulated | 5.39e-05 |
| 40. | UBXN7-AS1 | 0.0002356 | Downregulated | 1.44e-01 |
| 41. | SPAG5-AS1 | 0.0002356 | Upregulated | 1.91e-26 |
| 42. | EFCAB14-AS1 | 0.0002356 | Upregulated | 2.38e-05 |
| 43. | VPS13A-AS1 | 0.0002356 | NA | NA |
| 44. | FOXD2-AS1 | 0.0002356 | Upregulated | 4.24e-71 |
| 45. | MID1IP1-AS1 | 0.0002356 | Downregulated | 1.17e-08 |
| 46. | EAF1-AS1 | 0.0002356 | Downregulated | 7.97e-01 |
| 47. | RRM1-AS1 | 0.0002356 | NA | 1.42e-04 |
| 48. | PRC1-AS1 | 0.0002356 | Upregulated | 1.16e-19 |
| 49. | CRTC3-AS1 | 0.0002356 | Upregulated | 1.47e-22 |
| 50. | DLGAP1-AS2 | 0.0002356 | Upregulated | 4.58e-10 |
| 51. | LINC00703 | 0.0008009 | NA | 3.85e-01 |
| 52. | LINC01280 | 0.0008009 | NA | NA |
| 53. | LINC00618 | 0.0008009 | Upregulated | 1.16e-03 |
| 54. | LINC01709 | 0.0008009 | NA | NA |
| 55. | LINC02228 | 0.0008009 | NA | 8.90e-06 |
| 56. | TSPEAR-AS2 | 0.0008009 | Downregulated | 3.19e-01 |
| 57. | PKP4-AS1 | 0.0008009 | Upregulated | 1.70e-21 |
| 58. | OPA1-AS1 | 0.0008009 | Upregulated | 2.34e-04 |
| 59. | NCBP2-AS1 | 0.0008009 | Upregulated | 3.26e-13 |
| 60. | DIAPH3-AS1 | 0.0008009 | NA | NA |
| 61. | TRPM2-AS | 0.0008009 | Upregulated | 2.57e-32 |
| 62. | IDI2-AS1 | 0.0008009 | Upregulated | 1.95e-04 |
| 63. | LIX1L-AS1 | 0.0008009 | Upregulated | 1.91e-08 |
| 64. | DEPDC1-AS1 | 0.0008009 | Upregulated | 6.23e-27 |
| 65. | APOBEC3B-AS1 | 0.0008009 | NA | NA |
| 66. | HMMR-AS1 | 0.0008009 | NA | 2.09e-13 |
| 67. | CNOT10-AS1 | 0.0008009 | NA | 4.50e-03 |
| 68. | ZBTB11-AS1 | 0.0008009 | Downregulated | 6.75e-02 |
| 69. | LINC00265 | 0.002510 | Upregulated | 4.54e-21 |
| 70. | LINC01521 | 0.002510 | Upregulated | 9.97e-11 |
| 71. | LINC02067 | 0.002510 | NA | 1.38e-04 |
| 72. | LINC02014 | 0.002510 | Upregulated | 3.82e-82 |
| 73. | LINC02503 | 0.002510 | NA | NA |
| 74. | LINC01842 | 0.002510 | Upregulated | 1.29e-22 |
| 75. | EML4-AS1 | 0.002510 | Downregulated | 1.27e-08 |
| 76. | ZMYM4-AS1 | 0.002510 | Downregulated | 3.98e-04 |
| 77. | MED8-AS1 | 0.002510 | Upregulated | 1.28e-27 |
| 78. | UBE2Q1-AS1 | 0.002510 | Upregulated | 1.10e-40 |
| 79. | ZBTB46-AS1 | 0.002510 | NA | 4.49-08 |
| 80. | ACBD3-AS1 | 0.002510 | Downregulated | 1.83e-02 |
| 81. | MAP3K20-AS1 | 0.002510 | Upregulated | 4.94e-08 |
| 82. | DENND6A-AS1 | 0.002510 | Downregulated | 6.67e-02 |
| 83. | DDX11-AS1 | 0.002510 | Upregulated | 3.35e-76 |
| 84. | CSRP3-AS1 | 0.002510 | Downregulated | 7.74e-02 |
| 85. | TMPO-AS1 | 0.002510 | Upregulated | 6.31e-53 |
| 86. | TBC1D3P1-DHX40P1 | 0.002510 | NA | 5.82e-02 |
| 87. | LINC02527 | 0.007222 | NA | NA |
| 88. | LINC00392 | 0.007222 | NA | NA |
| 89. | LINC01122 | 0.007222 | Downregulated | 9.75e-01 |
| 90. | LINC01096 | 0.007222 | NA | 2.98e-12 |
| 91. | LINC02582 | 0.007222 | NA | 2.58e-02 |
| 92. | LINC02035 | 0.007222 | Downregulated | 1.77e-15 |
| 93. | KTN1-AS1 | 0.007222 | Upregulated | 9.88e-37 |
| 94. | SLC25A5-AS1 | 0.007222 | Upregulated | 2.72e-08 |
| 95. | DNMBP-AS1 | 0.007222 | Upregulated | 7.71e-19 |
| 96. | BZW1-AS1 | 0.007222 | Upregulated | 5.97e-07 |
| 97. | SGO1-AS1 | 0.007222 | NA | 3.91e-03 |
| 98. | ELFN1-AS1 | 0.007222 | Upregulated | 5.19e-15 |

* CARS-AS1 and PCBP2-OT1 not found in UALCAN

**Supplementary Table 17: hsa-let-7b-5p and MELK associated lncRNAs**

| **Index** | **Name** | **Correlation with hsa-let-7b-5p** | **Correlation with MELK** | **Correlation with FOXM1** |
| --- | --- | --- | --- | --- |
| 1. | GTF3C2-AS1 | -0.196 | 0.149 | 0.106 |
| 2. | NFYC-AS1 | -0.108 | -0.037 | -0.128 |
| 3. | LINC00449 | 0.022 | 0.106 | 0.153 |
| 4. | PITRM1-AS1 | -0.173 | 0.073 | -0.008 |
| 5. | SMG7-AS1 | -0.046 | NA | 0.034 |
| 6. | VPS9D1-AS1 | -0.267 | 0.225 | 0.295 |
| 7. | ODF2-AS1 | -0.097 | -0.005 | -0.057 |
| 8. | STARD7-AS1 | -0.149 | 0.110 | 0.198 |
| 9. | PRMT5-AS1 | -0.229 | 0.116 | 0.050 |
| 10. | LIX1-AS1 | 0.080 | -0.040 | -0.175 |
| 11. | IGBP1-AS2 | 0.016 | -0.030 | -0.110 |
| 12. | SPAG5-AS1 | -0.282 | 0.092 | 0.041 |
| 13. | EFCAB14-AS1 | -0.039 | -0.096 | -0.141 |
| 14. | FOXD2-AS1 | -0.181 | 0.189 | 0.254 |
| 15. | PRC1-AS1 | -0.230 | 0.175 | 0.182 |
| 16. | CRTC3-AS1 | 0.014 | 0.032 | -0.015 |
| 17. | DLGAP1-AS2 | -0.178 | 0.119 | 0.180 |
| 18. | LINC00618 | -0.077 | 0.151 | 0.141 |
| 19. | PKP4-AS1 | -0.039 | 0.050 | 0.023 |
| 20. | OPA1-AS1 | -0.112 | 0.148 | 0.062 |
| 21. | NCBP2-AS1 | -0.221 | 0.038 | 0.027 |
| 22. | TRPM2-AS | -0.244 | 0.225 | 0.290 |
| 23. | IDI2-AS1 | -0.211 | 0.131 | 0.123 |
| 24. | LIX1L-AS1 | -0.067 | -0.112 | -0.175 |
| 25. | DEPDC1-AS1 | -0.107 | 0.517 | 0.471 |
| 26. | LINC00265 | -0.258 | 0.066 | 0.157 |
| 27. | LINC01521 | 0.003 | 0.205 | 0.254 |
| 28. | LINC02014 | 0.027 | -0.029 | 0.010 |
| 29. | LINC01842 | -0.032 | 0.195 | 0.150 |
| 30. | UBE2Q1-AS1 | -0.176 | 0.018 | -0.029 |
| 31. | MAP3K20-AS1 | -0.055 | 0.153 | 0.113 |
| 32. | DDX11-AS1 | -0.317 | 0.464 | 0.467 |
| 33. | TMPO-AS1 | -0.277 | 0.626 | 0.659 |
| 34. | KTN1-AS1 | -0.160 | 0.326 | 0.305 |
| 35. | SLC25A5-AS1 | -0.074 | -0.056 | -0.119 |
| 36. | DNMBP-AS1 | 0.159 | -0.130 | -0.167 |
| 37. | ELFN1-AS1 | -0.249 | 0.282 | 0.380 |

* PABPC4-AS1, EXOSC10-AS1, H2AZ1-DT, SREBF2-AS1, LINC01357, EMC1-AS1, LLPH-DT, MED8-AS1, and BZW1-AS1 not found in ENCORI

**Supplementary Table 18: Prognostic Significance of lncRNAs in Lung Cancer**

| **Index** | **Name** | **HR** | **CI** | **P Value** |
| --- | --- | --- | --- | --- |
| **OS** | | | | |
| 1. | VPS9D1-AS1 | 0.99 | 0.85-1.14 | 0.87 |
| 2. | DDX11-AS1 | 1.12 | 0.97-1.3 | 0.12 |
| 3. | TMPO-AS1 | 1.5 | 1.29-1.74 | 7.2e-08 |
| 4. | ELFN1-AS1 | 1.07 | 0.92-1.24 | 0.36 |
| **OS + LUAD** | | | | |
| 1. | TMPO-AS1 | 2.16 | 1.69-2.76 | 4.1e-10 |
| **OS + LUAD + Smokers** | | | | |
| 1. | TMPO-AS1 | 2.34 | 1.41-3.88 | 0.00066 |
| **OS + LUAD + Smokers + Female** | | | | |
| 1. | TMPO-AS1 | 3.13 | 1.18-8.29 | 0.016 |

* PRC1-AS1, NCBP2-AS1, TRPM2-AS, and IDI2-AS1 not found in KM Plotter

**Supplementary Table 19: Correlation of Cell cycle regulator with MELK, FOXM1, hsa-let-7b-5p, TMPO-AS1**

| **S.NO.** | **Cell cycle Regulators** | **FOXM1**  **(R-value)** | **hsa-let-7b-5p**  **(R-value)** | **TMPO-AS1**  **(R-value)** |
| --- | --- | --- | --- | --- |
| 1 | CDK1 | 0.775 | -0.319 | 0.626 |
| 2 | CCNB1 | 0.795 | -0.378 | 0.581 |
| 3 | PLK1 | 0.891 | -0.382 | 0.638 |
| 4 | CDC25C | 0.791 | -0.382 | 0.622 |

| **S.NO.** | **EMT marker** | **FOXM1**  **(R-value)** | **hsa-let-7b-5p**  **(R-value)** | **TMPO-AS1**  **(R-value)** |
| --- | --- | --- | --- | --- |
| 1. | TWIST1 | 0.307 | -0.062 | 0.230 |
| 2. | SNAI1 | 0.383 | -0.148 | 0.172 |

**Supplementary Table 20: Correlation of EMT marker with FOXM1, hsa-let-7b-5p, TMPO-AS1**
**Supplementary Table 21: Primer sequences**

| **Primer** | **Sense (5' to 3')** | **Anti-sense (5' to 3')** |
| --- | --- | --- |
| MELK | CACCTCACGGCTACCTAT | TATCACTTGCGGTCACAT |
| GAPDH | AATCCCATCACCATCTTCCAG | AAATGAGCCCCAGCCTTC |
| U6 | CTCGCTTCGGCAGCACA | AACGCTTCACGAATTTGCGT |
| TMPO-AS1 | CAGTTTAAAAGGCGCTGGGG | CGCACAAAAGCAGTACGACC |
| hsa -let-7b-5p | CCAGCTGGGTGAGGTAGTAGGTTGT | CTGGAGCTAGTTTCGTCGTAGGG |
| hsa -let-7b-5p  (RT) | CTCAACTGGAGCTAGTTTCGTCGTAGGGCAGTTGAGAACCACAC | |
| U6 (RT) | CTCAACTGGTGTCGTGGAGTCGGCAATTCAGTTGAGAAAAATATG | |
